# Pattern-induced local symmetry breaking in active-matter systems

**DOI:** 10.1073/pnas.2010302117

**Published:** 2020-11-30

**Authors:** Jonas Denk, Erwin Frey

**Affiliations:** ^a^Arnold Sommerfeld Center for Theoretical Physics, Department of Physics, Ludwig-Maximilians-Universität München, D-80333 München, Germany;; ^b^Center for NanoScience, Department of Physics, Ludwig-Maximilians-Universität München, D-80333 München, Germany;; ^c^Department of Physics, University of California, Berkeley, CA 94720;; ^d^Department of Integrative Biology, University of California, Berkeley, CA 94720

**Keywords:** active-matter theory, pattern formation, emergent symmetries, pattern coexistence

## Abstract

The emergence of macroscopic order and patterns is a central paradigm in systems of (self-)propelled agents and a key component in the structuring of many biological systems. The relationships between the ordering process and the underlying microscopic interactions have been extensively explored both experimentally and theoretically. While emerging patterns often show one specific symmetry (e.g., nematic lane patterns or polarized traveling flocks), depending on the symmetry of the alignment interactions patterns with different symmetries can apparently coexist. Indeed, recent experiments with an actomysin motility assay suggest that polar and nematic patterns of actin filaments can interact and dynamically transform into each other. However, theoretical understanding of the mechanism responsible remains elusive. Here, we present a kinetic approach complemented by a hydrodynamic theory for agents with mixed alignment symmetries, which captures the experimentally observed phenomenology and provides a theoretical explanation for the coexistence and interaction of patterns with different symmetries. We show that local, pattern-induced symmetry breaking can account for dynamically coexisting patterns with different symmetries. Specifically, in a regime with moderate densities and a weak polar bias in the alignment interaction, nematic bands show a local symmetry-breaking instability within their high-density core region, which induces the formation of polar waves along the bands. These instabilities eventually result in a self-organized system of nematic bands and polar waves that dynamically transform into each other. Our study reveals a mutual feedback mechanism between pattern formation and local symmetry breaking in active matter that has interesting consequences for structure formation in biological systems.

## Significance

Propelled agents that align their orientations upon collisions can give rise to macroscopic order. Depending on the details of the agents’ collisions, this ordering process can lead to spatiotemporal patterns, including traveling polar waves and nematic lanes. Recent experiments suggest that such patterns can also coexist and dynamically interconvert. Here we propose a general mechanism for such a phenomenology, which is based on patterned-induced local symmetry breaking. We show that macroscopic order in active-matter systems is linked to spatiotemporal density patterns, which in turn can lead to local symmetry breaking if the agent density exceeds the relevant threshold values. Our study sheds light on the principles of pattern formation and the emergence of symmetry in biological active-matter systems.

Any theory for systems of (self-)propelled agents must be based on assumptions regarding the agents’ propulsion mechanism as well as their interactions. One of the central insights in active-matter theories is that interactions that align the agents’ orientations—even if they are short ranged—can lead to the formation of macroscopic order already in dilute systems in two dimensions (1–4). Close to the onset of macroscopic order, both experiments with (self-)propelled agents and theoretical studies quite generally observe phase separation into high-density ordered clusters and a low-density disordered background, rather than spatially uniform long-range order (3–5). Hence, symmetry breaking in active-matter systems seems to be inextricably linked to formation of patterns.

**Fig. 1.**
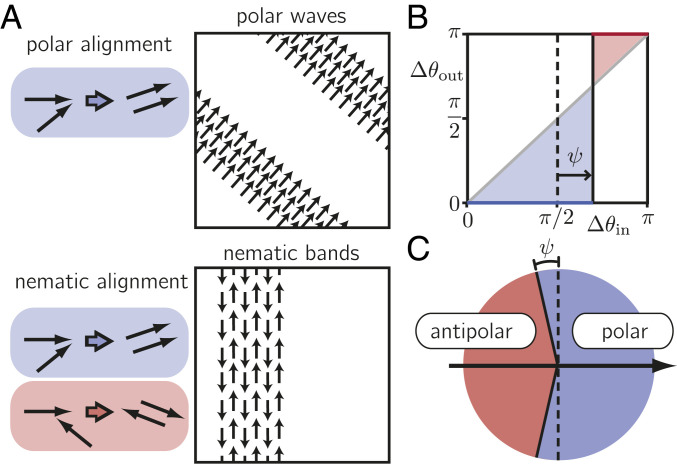
Symmetries in active matter. (*A*) In models with fully polar alignment, polar agents assume the same propulsion direction upon alignment. For fully nematic alignment, particles assume the same or opposing propulsion directions, depending on whether they collide at an acute or an obtuse angle, respectively. Polar (nematic) alignment interactions enable macroscopic polar (nematic) order. Beyond the onset of order, the system evolves into propagating wave patterns or nematic bands, depending on the symmetry of the alignment interaction. While the agents’ propulsion directions are oriented orthogonal to the wave front in polar wave patterns, in nematic bands they are aligned along the bands themselves. (*B*) “Collision statistics” for the binary collision rule with polar bias ψ. $\Delta \theta_{\text{out}}$ denotes the angle differences between pairwise particle velocities after the collision and is either 0 or π, depending on whether the agents’ angle difference before the collision, $\Delta \theta_{\text{in}}$, is smaller or larger than π/2+ψ, respectively. (*C*) Illustration of our generalized collision assumption. The black arrow indicates the precollision orientation of a reference polar agent. Alignment with a second agent is polar if the propulsion of the second particle lies in the blue shaded angular range and antipolar in the red shaded angular range.

In theoretical approaches, the symmetry of the macroscopic order and the corresponding patterns is typically dictated a priori by the assumed microscopic symmetry of the specific active-matter model under consideration (4) (Fig. 1*A*): Models with polar interaction symmetry exhibit propagating polar waves (6–10), in which the agents’ directions of propulsion point orthogonal to the wave front. On the other hand, in models with nematic interaction symmetry the agents form bands (lanes) within which the agents are (preferentially) oriented in parallel along the band (11–13). Thus, in all of these theoretical models, the choice of the underlying microscopic interaction symmetry largely determines the model’s phenomenology. This should be seen in light of the observation that in nature or in the laboratory microscopic details of the agent’s propulsion mechanism and interactions are often unclear or essentially inaccessible. Moreover, these properties of the agents might not even be inherent features (traits) characterized by a fixed set of parameters, but could in principle dynamically depend on the emergent collective behavior of the agents, as suggested for animal herds (14) or chemical active systems (15, 16).

Recently, experimental studies of actomyosin motility assays reported coexistence of polar and nematic patterns, with actin filaments dynamically cycling between polar waves and nematic band patterns (17). Supported by large-scale computer simulations—emulating the microscopic features of the observed collision statistics—these authors concluded that in this particular case the symmetry of the self-organized patterns is not determined a priori by the symmetry of the pairwise interaction between particles, but is itself an emergent phenomenon of the many-body system (17). What then is the mechanism underlying this startling phenomenon?

Agent-based simulations indicate that both nematic and polar-ordered clusters can arise and even coexist when the microscopic alignment between agents is predominantly nematic with a polar contribution, due to either the interactions between extended rods (5) or memory in the orientational noise (18). Furthermore, analytical studies that account for contributions from polar and nematic alignment due to the agents’ extensions (19–22) or assumed mixed alignment symmetries (23–25) identified distinct regimes of either nematic or polar patterns and even indicate coexistence of spatially separated polar and nematic patterns (22). However, cycling and transformations between patterns of different symmetries as observed in ref. 17 were not reported and the theoretical mechanism behind this coexistence is poorly understood (4, 17, 26). Specifically, neither kinetic nor continuum hydrodynamic approaches have so far been able to reproduce or elucidate this phenomenology (4, 26).

While theoretical approaches with mixed microscopic alignment symmetries have considered alignments that depend on interparticle distance (23), chance (24), or particle species (25), the computational analysis in ref. 17 suggested that the emergence of dynamic coexistence critically depends on the simultaneous presence of polar and nematic contributions in the binary collision statistics. Here, motivated by the results in ref. 17, we propose a kinetic theory for a dilute system of propelled particles with tunable “binary collision statistics” (Fig. 1*B*). Specifically, we employ a kinetic Boltzmann approach (27) where particles undergo binary collisions that lead to nematic alignment with a small (tunable) polar bias (Fig. 1*C*).

For both vanishing and fully polar bias, our model recovers the well-studied scenarios of purely nematic (13) and purely polar (9, 10) interaction symmetry, respectively. Interestingly, for an intermediate polar bias, our model features a transition from macroscopic nematic order at intermediate densities to macroscopic polar order at high densities. In a regime characterized by intermediate polar bias and intermediate densities, we observe that patterns of polar and nematic symmetry coexist and are dynamically interconvertible, which is reminiscent of the observations in ref. 17. Based on a combination of stability analyses and numerical simulations we argue that such coexistence depends on the inextricable link between symmetry breaking and pattern formation. For instance, while the system forms nematic bands in a density regime that leads to symmetry breaking toward macroscopic nematic order, the density at the core of these bands increases and eventually exceeds the threshold value for a transition from macroscopic nematic to macroscopic polar order. This spatially local crossing of a critical value in the particle density—a control parameter—triggers local symmetry breaking, which induces the self-organized formation of polar waves.

To substantiate this hypothetical mechanism as a general mechanism for the coexistence of polar and nematic patterns in active-matter systems, we study simplified hydrodynamic equations which capture pattern formation in a nematic phase as well as a transition from macroscopic nematic to polar symmetry for high densities. Indeed, like our kinetic Boltzmann approach, our hydrodynamic theory exhibits a regime of coexisting polar wave and nematic band patterns.

Our study thus reveals an interesting mutual feedback between pattern formation and macroscopic symmetry breaking in active matter. This feedback occurs because the particle density, which shows pattern formation in active systems, is at the same time a control (bifurcation) parameter for the macroscopic symmetry of the system. This twofold role of the particle density transforms symmetry breaking in active systems from an ordering phenomenon under the control of a global parameter into a self-organization phenomenon with a local interplay between pattern formation and symmetry breaking. We argue that this interplay represents a fairly general mechanism that allows macroscopic symmetries to be an emergent property in themselves, rather than being imposed directly by microscopic interaction rules.

## Results

### Kinetic Boltzmann Approach with Polar and Nematic Contributions.

Our starting point for a mesoscopic theory of aligning agents is the kinetic Boltzmann equation (27). It describes the temporal evolution of the one-particle distribution function $f\left(r,\theta ,t\right)$ for the position $r\in R^{2}$ and the orientation θ∈[0,2π) of self-propelled particles that undergo binary aligning collisions in a dilute (dry) system (27). It reads

$$\partial_{t}f\left(r,\theta ,t\right)+v_{0}\;e_{\theta }\cdot \partial_{r}f\left(r,\theta ,t\right)\;=I_{\text{diff}}\left[f\right]+I_{\text{coll}}\left[f,f\right],$$ [1]

where $v_{0}$ denotes the constant speed of the active particles, $e_{\theta }$ is a unit vector pointing along direction θ, and the terms $I_{\text{diff}}\left[f\right]$ and $I_{\text{coll}}\left[f,f\right]$ describe diffusion of individual particles and collisions between particles, respectively (*SI Appendix*, section 1). In more detail, spherical particles (with diameter d) are assumed to move ballistically with constant speed $v_{0}$ along their orientations θ and can change their orientation by diffusion as well as by local binary collision. Diffusion is modeled by a shift in a particle’s orientation from θ to θ+η at a rate λ, where we assume η to be a Gaussian-distributed random variable with standard deviation σ. Binary collisions between particles are emulated through “alignment rules” (Fig. 1 *B* and *C*), with an additive random contribution also drawn from a Gaussian distribution with a standard deviation $\sigma^{\prime }$; in the following we set $\sigma^{\prime }=\sigma$ for simplicity. In the context of the kinetic Boltzmann approach, a fully nematic interaction rule dictates that particles that collide at an acute angle adopt their average orientation (polar alignment), while particles colliding at an obtuse angle also align, but with opposite orientations (antipolar alignment). For fully polar alignment, particles adopt their average orientation irrespective of their precollision angle (Fig. 1).

The dynamics of orientational order in the kinetic Boltzmann approach are most conveniently studied by exploiting the fact that the polar vector P and nematic tensor Q can be expressed in terms of the Fourier modes $f_{k}\left(r,t\right)=\int_{-\pi }^{\pi }\;d\theta \;e^{i\theta k}f\left(r,\theta ,t\right)$ of the one-particle distribution function:

$$\rho P=\left(\begin{array}{c} Re\left[f_{1}\right]\\ Im\left[f_{1}\right] \end{array}\right),\;\rho Q=\frac{1}{2}\left(\begin{array}{cc} Re\left[f_{2}\right] & Im\left[f_{2}\right]\\ Im\left[f_{2}\right] & -Re\left[f_{2}\right] \end{array}\right).$$ [2]

Furthermore, the local particle density $\rho \left(r,t\right)$ is given by the k=0 mode: $\rho \left(r,t\right)=f_{0}\left(r,t\right)$. The dynamics of $f_{k}\left(r,t\right)$ read

$$\begin{array}{ll} & \partial_{t}f_{k}+\frac{v_{0}}{2}\left[\partial_{x}\left(f_{k+1}+f_{k-1}\right)-i\partial_{y}\left(f_{k+1}-f_{k-1}\right)\right]\\ & \;=-\lambda \left(1-e^{-\frac{1}{2}k\sigma^{2}}\right)f_{k}+\overset{\infty }{\underset{n=-\infty }{\sum }}\;I_{n,k}f_{n}f_{k-n}. \end{array}$$ [3]

Explicit expressions for the collision coefficients $I_{n,k}$ can be found in *SI Appendix*, section 1. For k=0, Eq. **3** yields the continuity equation $\partial_{t}\rho \left(r,t\right)=-\;v_{0}\nabla \cdot \left(\rho P\right)$.

Solutions of Eq. **3** for fully polar (9, 10) or fully nematic (13) alignment rules show a transition from disorder, i.e., vanishing polar and nematic order, to nonzero polar or nematic order, respectively, for sufficiently high densities or low noise level σ. Close to the onset of order, it predicts the formation of patterns, consistent with experimental observations and numerical simulations (3, 28). The kinetic Boltzmann equation thus serves as a useful basis for a qualitative study of the phenomenology of dilute systems of self-propelled particles.

Recent experimental results from the actin motility assay and corresponding simulation results from agent-based models (17) strongly suggest that the relative weights of polar and nematic contributions to the binary collision statistics are critical for the self-organization of spatiotemporal patterns. As a minimal extension of fully polar or nematic alignment rules (27), we propose a collision rule with a small tunable polar bias. Specifically, we assume that colliding particles align in a polar manner when their velocities form an angle difference smaller than $\frac{\pi }{2}+\psi$ with $\psi \in \left[0,\frac{\pi }{2}\right]$ and align antipolar otherwise (Fig. 1 *B* and *C*). The parameter ψ thus characterizes the polar bias, where for ψ=0 and $\psi =\frac{\pi }{2}$ the collision rule reduces to fully nematic and fully polar collisions, respectively. It is convenient to rescale time, space, and density such that $v_{0}=\lambda =d=1$. Then, the only remaining free parameters are the noise amplitude σ, the polar bias ψ, and the mean particle density $\bar{\rho }=\frac{1}{A}\int_{A}\;dr\int_{-\pi }^{\pi }\;d\theta \;f\left(r,\theta ,t\right)$ measured in units of $\lambda /\left(dv_{0}\right)$, i.e., the number of particles found within the area traversed by a particle between successive diffusion events.

### Mean-Field Phase Diagram.

**Fig. 2.**
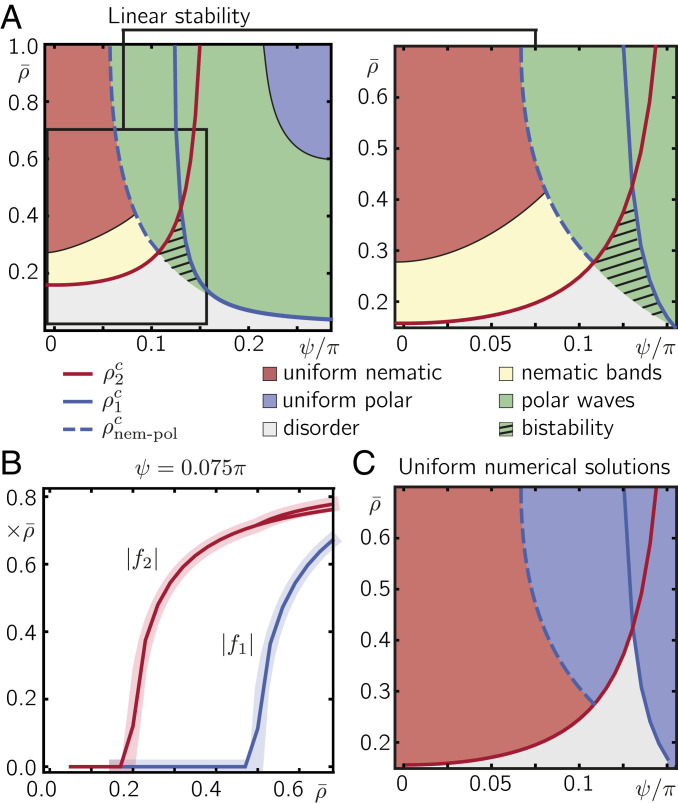
Uniform solutions and linear stability. (*A*) Regimes and linear stability of spatially uniform solutions for Eq. **3** in angular Fourier space with a truncation at $k_{c}=10$. Solid lines denote the critical transition densities $\rho_{2}^{c}$ and $\rho_{1}^{c}$ from a disordered solution to nematic and polar order, respectively. Our analysis reveals another transition from nematic order to polar order at intermediate polar bias (blue dashed line). (*B*) Spatially uniform solutions for $f_{1}$ and $f_{2}$ at ψ=0.075π calculated from the truncated Boltzmann equation in angular Fourier space (solid lines) compared to spatially uniform solutions of the Boltzmann equation in real space (Eq. **1**, shaded lines) calculated using the generalized SNAKE algorithm. (*C*) Phase portrait of spatially uniform solutions using the generalized SNAKE algorithm (29). The numerical solutions used a single lattice point starting at a disordered state with small fluctuations in the angular distribution. The noise value was fixed to σ=0.2.

Since the collision coefficients $I_{n,k}$ are zero for k=0, Eq. **3** possesses a spatially uniform solution with vanishing order, i.e., $f_{k}=0$ for |k|>0, and uniform density $f_{0}=\bar{\rho }$. To linear order, a small perturbation $\delta f_{k}$ of this disordered state evolves according to $\partial_{t}\delta f_{k}\left(t\right)=\mu_{k}\left(\bar{\rho },\sigma ,\psi \right)\delta f_{k}$ with the growth rate $\mu_{k}\left(\bar{\rho },\sigma ,\psi \right)=\left(I_{0,k}+I_{k,k}\right)\bar{\rho }-\lambda \left(1-e^{-\frac{1}{2}k\sigma^{2}}\right)$. The zeros of these growth rates, $\mu_{k}\left(\rho_{k}^{c},\sigma ,\psi \right)=0$, mark the critical densities $\rho_{k}^{c}\left(\sigma ,\psi \right)$ above which the mode k grows exponentially. While previous studies have focused on the onset of order for fully polar and nematic interactions as a function of the density $\bar{\rho }$ and noise amplitude σ (27), in the following we keep the noise level constant, σ=0.2, and focus on the onset of order as a function of the polar bias ψ. Fig. 2*A* shows the critical densities $\rho_{1}^{c}\left(\psi \right)$ and $\rho_{2}^{c}\left(\psi \right)$ for the onset of polar and nematic order, which have been calculated by setting $\mu_{1}$ and $\mu_{2}$ to zero and numerically solving for $\bar{\rho }$, respectively. For small and large polar bias, only the growth rate for k=2 or k=1, respectively, changes sign, indicating that there are transitions from a disordered state to a state with either nematic order (for small polar bias ψ) or polar order (for large polar bias ψ). In contrast, for intermediate polar bias, the transition densities $\rho_{1}^{c}\left(\psi \right)$ and $\rho_{2}^{c}\left(\psi \right)$ cross, implying that there is a regime in the ($\bar{\rho }$, ψ) phase diagram where the disordered state is linearly unstable under both polar and nematic perturbations. For a more detailed discussion of the transition densities and the linear stability analysis see *SI Appendix*, section 1.

After identifying the parameter regimes where the spatially uniform disordered solutions become unstable, we now determine the stable, spatially uniform, ordered solutions of Eq. **3** in these regimes. As this is no longer feasible analytically (due to the infinite sum in Eq. **3**), we resort to approximate solutions, exploiting the fact that even above the ordering transition, modes with sufficiently large |k| are still negligible (10, 30). To this end, following ref. 30, we set all spatial derivatives and all Fourier modes $f_{k}$ beyond a certain cutoff $k_{c}$ in Eq. **3** to zero and numerically solve the ensuing equations for all remaining modes with $|k|\leq k_{c}$ (see *SI Appendix*, section 1A for details of our linear stability analyses). We then performed a linear stability analysis of the resulting spatially uniform solutions against uniform as well as nonuniform (wave-like) perturbations. The directions of spatial perturbations were varied to probe for instabilities perpendicular and parallel to the orientation of the spatially uniform (polar and nematic) order parameters. Based on this analysis we identified the type of order exhibited by spatially uniform solutions, as well as their stability against wave-like perturbation for different values of the average density $\bar{\rho }$ and polar bias ψ (Fig. 2*A*).

Above the critical transition densities $\rho_{2}^{c}\left(\psi \right)$ and $\rho_{1}^{c}\left(\psi \right)$, we indeed find spatially uniform solutions with nonzero nematic and polar order, respectively, as suggested by the linear stability analysis of the disordered state. In addition, within these regimes, we identify subregimes in which the respective spatially uniform solutions are unstable under spatial perturbations, suggesting the formation of spatially nonuniform patterns (indicated by yellow and green in Fig. 2*A*, denoting nematic and polar patterns, respectively). More precisely, we find that right above the transition density to nematic order, $\rho_{2}^{c}$, spatially uniform nematic order is unstable against wave-like perturbations perpendicular to the nematic order, which suggests formation of nematic band patterns. Furthermore, in a subregime of polar order, uniform polar order is unstable against spatial perturbations parallel to the orientation of polar order, which suggests the emergence of traveling polar waves. The prediction of nematic bands and polar waves for small and large polar bias is in accordance with previous studies on systems with either fully nematic or polar interaction symmetries, respectively (27). Interestingly, for sufficiently large polar bias (ψ≳0.05π) and high enough densities ($\bar{\rho }>\rho_{2}^{c}$) we identify a transition that has not been observed in previous studies with fully nematic or polar alignment: As the density is increased above $\rho_{\text{nem}-\text{pol}}^{c}\left(\psi \right)$ (indicated by the dashed line in Fig. 2*A*), there is a direct transition from solutions with uniform nematic order to solutions with uniform polar order.

Furthermore, within the regime where the disordered solution is linearly stable and for densities right below the intersection of $\rho_{1}^{c}$ and $\rho_{2}^{c}$, our analysis reveals a regime of bistability (black hatched regime in Fig. 2*A*). Here, we find a linearly stable disordered solution as well as a uniform polar solution, which is linearly stable against spatially uniform perturbations, but linearly unstable against spatially nonuniform perturbations with wave vector orthogonal to the polar order. This implies bistability; i.e., depending on the initial conditions the ensuing collective state (attractor of the dynamics) is either a uniformly disordered state or a polar wave state. Within the range of polar bias values that limit the regime of bistability (approximately between ψ/π=0.1 and ψ/π=0.15 in Fig. 2*A*), the transition line marked by $\rho_{1}^{c}$ can be understood as a “spinodal”; i.e., for densities above $\rho_{1}^{c}$ polar patterns form spontaneously. In the same sense, the line demarcating the bistable region can be considered as a “binodal”; i.e., for densities above this line (but below $\rho_{1}^{c}$) polar patterns are metastable and require a large enough perturbation of the disordered state to be able to form (see *SI Appendix*, section 1A and Fig. S1 for a more detailed discussion). This bistability between the uniformly disordered state and polar waves is an interesting topic in itself. We defer the study of this bistability regime to future work and focus here on the transition from nematic to polar order.

To independently test the predictions of our approximate solutions and linear stability analyses, we numerically solved the Boltzmann equation, Eq. **1**, in real space using a finite difference method; for details on the SNAKE (solving numerically active kinetic equations) algorithm (29) see *SI Appendix*, section 1B. Fig. 2*C* shows the phase diagram obtained from the numerical solution for spatially uniform systems, which is in excellent agreement with the approximate solutions of our spectral analysis (Fig. 2*A*); see also Fig. 2*B*.

### Pattern Formation Leads to Dynamic Transformations between Nematic and Polar Symmetries.

**Fig. 3.**
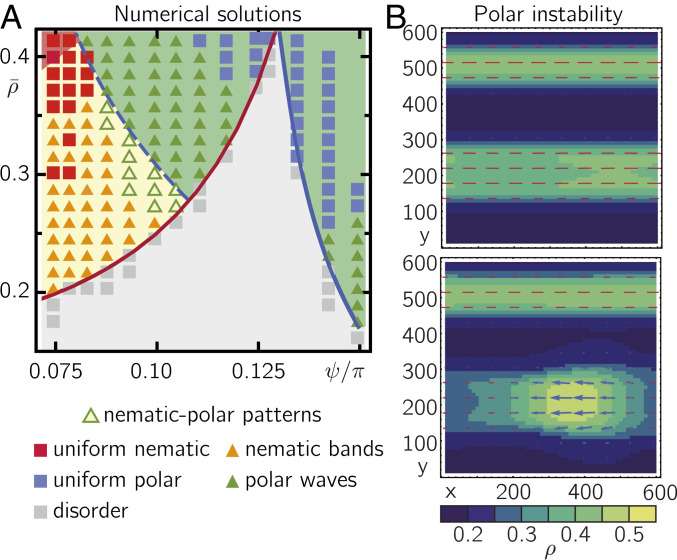
Dynamic transformations between nematic and polar patterns. (*A*) Numerical solutions of Eq. **1** (indicated by symbols) display regimes of uniform nematic and polar order as well as nematic band and traveling-wave patterns (shaded background colors as in Fig. 2*A*). The green and yellow shades denote regimes in which linear stability analysis predicts polar and nematic patterns, respectively (regimes in in Fig. 2 *A* and *C*). In addition to the states predicted by linear stability analysis of uniform solutions (Fig. 2), we find dynamic transformations between patterns of nematic to polar patterns below $\rho_{\text{nem}-\text{pol}}^{c}$. (*B*) Snapshots of the numerical solution of the Boltzmann equation in the regime where polar bands and nematic lanes interconvert into each other (nematic–polar patterns) shortly before (*Top*) and after (*Bottom*) a local instability within the core of a nematic band (ρ=0.285, ψ/π=0.1). Red bars and blue arrows indicate the orientation and strength of local nematic and polar order, respectively. The color denotes the local density. All numeric solutions were initialized with a uniform disordered state with small random fluctuations. For details on the numerical solutions see *SI Appendix*, section 1B.

Next, we study the full spatiotemporal dynamics of the kinetic Boltzmann equation, Eq. **1**, especially in those regimes where our stability analysis predicts spatially uniform solutions to be linearly unstable. To this end, we numerically solved Eq. **1** using the SNAKE algorithm (29) for a spatially extended system in a broad parameter regime of the global density and polar bias. The results are shown as symbols in Fig. 3*A* against the background of the predictions from the linear stability analysis (shaded) (Fig. 2*A*). For vanishing and small polar bias, we observe nematic bands at densities right above $\rho_{2}^{c}\left(\psi \right)$ and uniform nematic states at higher densities (orange and red symbols in Fig. 3*A*, respectively; *SI Appendix*, section 1B and Fig. S2*A*). For larger polar bias, we find regimes of traveling-wave solutions and spatially uniform polar-ordered states (green and blue symbols in Fig. 3*A*, respectively; *SI Appendix*, section 1B and Fig. S2*B*). Consistent with linear stability analysis (Fig. 2*A*), our numerical solution yields patterns for density values close above the transition densities $\rho_{1}^{c}$ and $\rho_{2}^{c}$. The regimes of patterns in our numeric solution are smaller than predicted by linear stability, probably due to finite size effects and spurious noise in the implementation of the SNAKE algorithm as suggested by earlier studies (30, 31).

Remarkably, we find patterns with polar order even for densities below $\rho_{\text{nem}-\text{pol}}^{c}$ (nematic–polar patterns in Fig. 3*A*). More precisely, we observe coexistence between polar and nematic bands, which dynamically interconvert into each other (Fig. 3*B*; *SI Appendix*, section 1B; and Movies S1–S3): Starting from a disordered state, the nematic order grows quickly and the system begins to exhibit high-density nematic band patterns (in line with linear stability analysis). While the ensuing average nematic order is approximately the same as that found for spatially uniform solutions of Eq. **3**, the local nematic order is much higher within the nematic bands and approaches zero in the disordered regions between the bands (*SI Appendix*, section 1B and Fig. S3). The local density in the center of a band is actually so high that it far exceeds the threshold density for the transition between nematic and polar order $\rho_{\text{nem}-\text{pol}}^{c}$. This suggests that within a band, purely nematic order eventually becomes unstable and polar order starts to emerge there. Indeed, we observe that after some time, polar order locally builds up in the nematic bands and subsequently leads to the formation of polar waves that propagate along the nematic band and whose density at the wave front exceeds $\rho_{\text{nem}-\text{pol}}^{c}$ (Fig. 3*B* and Movies S1–S3). As a polar wave front forms within a nematic band, this band locally broadens and density is distributed from the high-density core of the band into the disordered regions adjacent to the band (Fig. 3*B* and Movies S1–S3). In the low-density regime adjacent to the nematic band, polar order eventually decays, and the density that had been redistributed to these areas condenses into existing (Movie S1) or new (Movie S3) nematic bands and the cycle starts anew. Initializing the system with different randomly disordered states, we find that the local polar instability eventually leads to various distinct types of spatiotemporal dynamics including polar waves within nematic bands (Fig. 3*B* and Movie S1), complete replacement of nematic bands by polar waves (Movie S2), and dynamic switching between nematic bands and polar waves (Movie S3).

The observation of different collective states, depending on the initial conditions, suggests that these states are metastable. This is in accordance with our numerical solutions which show that at the transition lines between regimes of vanishing polar order and patterns with polar order (i.e., regimes of “polar waves” and “nematic–polar patterns” in Fig. 3*A*) the system undergoes discontinuous transitions (*SI Appendix*, section 1B and Fig. S4). It will be interesting to explore the nature of the transitions between regimes of nematic and polar order as well as the formation and dynamics of the metastable states more closely in follow-up studies.

In summary, for low and high polar bias our kinetic Boltzmann approach is consistent with the classical conception of self-propelled particle systems with predominantly nematic or polar symmetry (4), including the formation of nematic band patterns and traveling polar waves at the onset of nematic and polar order, respectively. For moderate polar bias, stability analysis uncovers an additional transition from nematic solutions to polar solutions at a density $\rho_{\text{nem}-\text{pol}}^{c}>\rho_{2}^{c}$. This transition gives rise to spatiotemporal dynamics that are not predicted by linear stability analysis of the spatially uniform solutions: The numerical solution of the Boltzmann equation reveals that the high-density core of nematic bands can locally cross the threshold density $\rho_{\text{nem}-\text{pol}}^{c}$, which favors the formation of polar waves. This instability eventually results in traveling-wave solutions, as well as more complex dynamics such as coexisting polar waves and nematic bands, and dynamic rearrangements of nematic bands.

Importantly, while our alignment rule contains contributions with both polar and nematic symmetry, the symmetries of the patterns are not already determined or apparent from the assumed alignment symmetry. Instead, the symmetries of the patterns are selected by and critically depend on the spatiotemporal dynamics of the system. We therefore argue that the symmetry of the spatiotemporal pattern is itself an emergent property in the following sense: The collective dynamics are based on a reciprocal feedback between pattern formation and local symmetry breaking due to the redistribution (accumulation) of mass (particle density).

### Hydrodynamic Equations Account for Coexisting Symmetries.

Complementary to kinetic approaches, hydrodynamic theories have served as a basis to study active-matter systems (2). They describe the system’s dynamics in terms of slow collective variables, such as the particle density and fields characterizing the macroscopic order. The underlying hydrodynamic equations are frequently—in the spirit of a Ginzburg–Landau approach—derived from symmetry arguments and small-amplitude expansions in the order of parameter fields and their gradients (1, 2, 32). The collective dynamics are then studied as a function of the coupling coefficients, which are considered to be free phenomenological parameters.

It is possible to link these phenomenological approaches to kinetic approaches based on the Boltzmann (27) and the Smoluchowski equation (21, 33) by using scaling assumptions for the order parameters and suitable truncation schemes for their underlying dynamic equations (27, 34). While this yields the structure of the hydrodynamic equations as well as explicit expressions for the coupling coefficients as functions of microscopic model parameters such as particle velocity and alignment noise, there are also some limitations, mainly due to potential ambiguities associated with the truncation scheme used; please refer to ref. 35 for a review and comparison of the different kinetic approaches. All these field theories, which are more amenable to theoretical analysis than the respective kinetic theories, have been successfully employed to reproduce and understand the rich phenomenology of active-matter systems (4, 36). In particular, starting from a kinetic Boltzmann equation, Peshkov et al. (13) derived hydrodynamic equations for purely nematic systems and found excellent agreement with previous microscopic agent-based simulations (12), including the formation of nematic band patterns close to the onset of nematic order. This success suggests that their approach can be generalized to systems with a small polar bias. Therefore, we adapt their analysis to our case and assume that, close to the onset of polar or nematic order, the respective order fields $f_{1}$ and $f_{2}$ and their temporal and spatial variations are small. Using their truncation scheme for higher modes, as explained in *SI Appendix*, section 2A, we arrive at closed equations for the dominant hydrodynamic fields,

$$\begin{array}{ll} \partial_{t}f_{1} & =-\left(\alpha_{0}+\alpha_{1}\rho \right)f_{1}+\alpha_{2}f_{1}^{*}f_{2}-\alpha_{3}|f_{2}{|}^{2}f_{1}\\ & \;-\frac{1}{2}\left(\nabla \rho +\nabla^{*}f_{2}\right)+\gamma_{1}f_{2}^{*}\nabla f_{2}, \end{array}$$ [4a]

$$\begin{array}{ll} \partial_{t}f_{2} & =\left(-\beta_{0}+\beta_{1}\rho \right)f_{2}+\beta_{2}f_{1}^{2}-\beta_{3}|f_{2}{|}^{2}f_{2}-\beta_{3}^{\prime }|f_{1}{|}^{2}f_{2}\\ & \;-\frac{1}{2}\nabla f_{1}+\gamma_{2}\nabla \nabla^{*}f_{2}-\gamma_{3}f_{1}^{*}\nabla f_{2}-\gamma_{4}\nabla^{*}\left(f_{1}f_{2}\right), \end{array}$$ [4b]

which are related to the polar and nematic order parameter through Eq. **2**. To simplify the notation, we used the definition $\nabla ≔\partial_{x}+i\partial_{y}$ and the asterisks denotes complex conjugation.

The derivation of Eq. **4** from the kinetic Boltzmann equation, Eq. **1**, also yields explicit expressions of the coefficients $\alpha_{i}$, $\beta_{i}$, and $\gamma_{i}$, which are determined by the angular diffusion term and combinations of the collision integrals $I_{n,k}$ introduced in Eq. **3**; for explicit expressions see *SI Appendix*, section 2A. However, using these coefficients to calculate the spatially uniform solutions of Eq. **4** as well as their linear stability, we find that the resulting phase diagram (*SI Appendix*, section 2A and Fig. S5) critically differs from the phase diagram obtained by solving the Boltzmann equation, Eq. **1** (Fig. 2*A*); see *SI Appendix*, section 2A for a more detailed discussion. Most importantly, it does not feature a secondary transition from a regime of nematic band patterns to polar-ordered solutions for increasing density as observed in Fig. 2*A* (blue dashed line). We attribute this difference to higher-order modes that become important for systems with polar bias but are disregarded in the truncation scheme adapted from active systems with purely nematic alignment (13). In fact, a numerical solution of Eq. **3**, which takes into account modes $f_{k}$ with |k| up to a certain $k_{h}$ and sets all modes with $|k|>k_{h}$ and their derivatives to zero (see *SI Appendix*, section 2B for details), shows that in the regime of nematic–polar patterns in Fig. 3*A* all modes eventually diverge, even for relatively large cutoff mode numbers $k_{h}≳12$ (*SI Appendix*, section 2B and Fig. S6).

In the following, we thus take a semiphenomenological approach which retains the structure of Eq. **4** but investigates the dynamics of this field theory for general coupling parameters. Similar to that for a Ginzburg–Landau theory for equilibrium phase transitions, there are some phenomenological constraints on the parameters. To reproduce a bifurcation from a disordered state to a nematic state at a critical density $\rho_{2}^{c}=\beta_{0}/\beta_{1}$, the parameters $\beta_{0}$ and $\beta_{1}$ need to be positive. The key phenomenological parameter in the hydrodynamic equations describing a (bilinear) coupling between nematic and polar fields is $\alpha_{2}$. Since in the absence of such a coupling, i.e., without a polar bias, the systems should only show nematic but no polar order, $\alpha_{0}$ and $\alpha_{1}$ are required to be positive. Given these constraints, the bilinear coupling term $\alpha_{2}f_{1}^{*}f_{2}$ in Eq. **4a** might still induce growth of polar order when the nematic amplitude $\left|f_{2}\right|$ is large enough such that the coupling term dominates the decay term $-\left(\alpha_{0}+\alpha_{1}\rho \right)f_{1}$. Finally, to ensure saturation of the amplitudes for both polar and nematic order, $\left|f_{1}\right|$ and $\left|f_{2}\right|$, the coefficients for the highest-order terms, i.e., $\alpha_{3}$ and $\beta_{3}$, have to be positive. The structure of the hydrodynamic equations, especially the density dependence of the first term in Eq. **4b** and the possibility of induced polar order due to a bilinear coupling term between polar and nematic order parameters, is consistent with hydrodynamic equations based on Smoluchowski approaches (37, 38). Taken together, this establishes Eq. **4** as a well-founded, semiphenomenological model for our further analysis. In the following, we use it to systematically study the effect of a varying polar–nematic coupling strength $\alpha_{2}$. For specificity, all other coefficients were fixed to values derived from the kinetic Boltzmann equation with fully nematic alignment and right above the transition to nematic order ($\rho =\bar{\rho }=0.16$ and σ=0.2). This choice naturally satisfies all of the abovementioned general conditions on the coefficients.

**Fig. 4.**
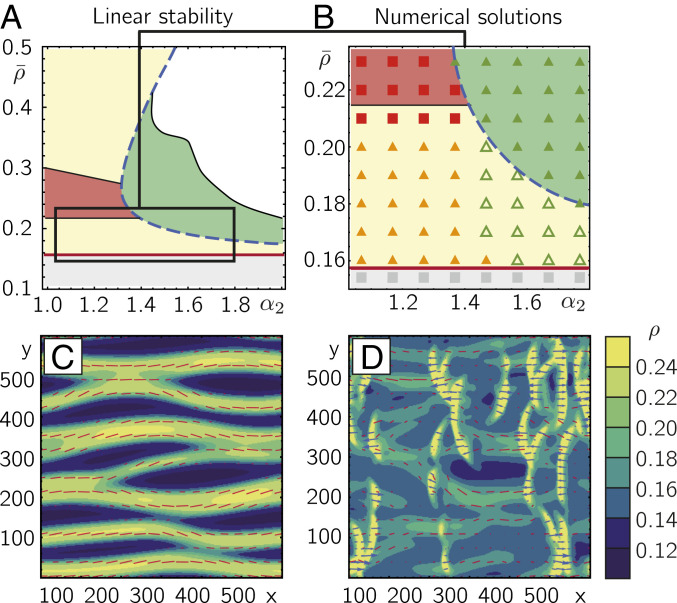
Cycling symmetries in the hydrodynamic approach. (*A*) Linear stability analyses of Eq. **4** yield a phase diagram with regimes of polar and nematic patterns that qualitatively resembles the phase diagram derived from the kinetic Boltzmann approach for a mixed collision rule (Fig. 2); colors denote regimes of different symmetries and patterns following the color legend in Fig. 2*A*. In particular, for densities close above $\rho_{2}^{c}$ (red solid line) linear stability predicts a phase of nematic band patterns (yellow area) and, for moderate coupling strengths $\alpha_{2}$ ($\alpha_{2}≳1.4$ in units of [density/time]), a transition between a regime of nematic bands and a regime of polar waves (green area) at a critical density $\rho_{\text{nem}-\text{pol}}^{\left(c,h\right)}$ (blue dashed line). When both $\alpha_{2}$ and $\bar{\rho }$ are large, there are no physical solutions (white area). For our numerical solutions of Eq. **4** we focus on the boxed regime close to the transition density $\rho_{2}^{c}$. (*B*) Numerical solutions of the hydrodynamic equations, Eq. **4** (denoted by the same symbols as in Fig. 3*A*), show the formation of nematic bands and polar waves, as well as coexistence patterns with transformations between nematic and polar patterns. (*C*) Snapshot of nematic band patterns shortly before the onset of local polar instabilities (red bars indicate orientation and strength of local nematic order). (*D*) At later times, polar instabilities lead to the formation of traveling-wave patterns with complex dynamics including coexistence of local nematic and polar ordered regions which interact and transform into each other. Blue arrows denote the strength and direction of polar order. (Parameters are $\alpha_{2}=1.5$ in units of [density/time], $\bar{\rho }=0.18$.) For details on the numerical solutions see *SI Appendix*, section 2C. In *C* and *D*, the color code denotes the local densities, and red bars and blue arrows show the orientation of local nematic and polar order, respectively (the length indicates their respective absolute amplitudes).

Fig. 4*A* shows the phase diagram as a function of the average density $\bar{\rho }$ and the polar–nematic coupling strength $\alpha_{2}$, obtained by calculating the spatially uniform solutions of Eq. **4** and their linear stability against uniform and nonuniform perturbations; for details see *SI Appendix*, section 2C. This phase diagram shares key qualitative similarities with the linear stability analysis of the kinetic Boltzmann equation (Fig. 2*A*). By construction, for average densities above $\rho_{2}^{c}$ the disordered solution is unstable and we find solutions with uniform nematic order (yellow and red areas in Fig. 4*A*). Right above $\rho_{2}^{c}$, uniform nematic solutions are stable against uniform perturbations, but unstable against nonuniform perturbations perpendicular to the orientation of nematic order, suggesting the formation of nematic band patterns (yellow area in Fig. 4*A*). For moderate coupling strengths $\alpha_{2}$ ($\alpha_{2}≳1.3$ in units of $\left[\text{density}/\text{time}\right]$) and densities above a certain density $\rho_{\text{nem}-\text{pol}}^{\left(c,h\right)}$, we find solutions with uniform polar order, which are stable against uniform perturbations, but unstable against nonuniform perturbations parallel to the orientation of polar order, suggesting the formation of polar wave patterns (green area in Fig. 4*A*). This is similar to our findings for the kinetic Boltzmann equation shown as a dashed line in Fig. 2*A*. When both $\alpha_{2}$ and $\bar{\rho }$ are large, there are no physical solutions (the polar order lies beyond the attainable density), indicating that the hydrodynamics equations are not adequate in these regimes (white area in Fig. 4*A*). For small $\alpha_{2}$ and large $\bar{\rho }$ we find reentrance into a regime of nematic patterns. In the following we disregard these regimes and focus on the regime close to the transition density $\rho_{2}^{c}$ (boxed regime in Fig. 4*A*).

To resolve the spatiotemporal dynamics of the polar and nematic modes beyond linear stability analysis, we numerically solved Eq. **4**, together with the continuity equation for the density, using XMDS2 software (39), a fast Fourier transform (FFT)-based spectral solver; see phase diagram in Fig. 4*B*. For low polar–nematic coupling strength, $\alpha_{2}≲1.4$, and slightly above the threshold density $\rho_{2}^{c}$, we find nematic band patterns as predicted by the linear stability analysis discussed above. For larger $\alpha_{2}$ and densities between $\rho_{2}^{c}$ and $\rho_{\text{nem}-\text{pol}}^{\left(c,h\right)}$, we find a dynamic that exhibits dynamic transitions between patterns of polar and nematic symmetry (Fig. 4 *C* and *D* and Movie S4) reminiscent of our observations in the kinetic Boltzmann approach (Fig. 3*B*). Starting from a disordered uniform state with superimposed small random fluctuations, the system first forms nematic bands as predicted by the linear stability analysis (Fig. 4*A*). Subsequently, we observe that at the core of these bands the local density increases and eventually exceeds $\rho_{\text{nem}-\text{pol}}^{\left(c,h\right)}$, which then should trigger a local instability toward polar order (*SI Appendix*, section 2C and Fig. S7). Indeed, the numerical solutions show the formation of traveling-wave patterns propagating along the nematic bands. These instabilities eventually result in intriguing spatiotemporal patterns of nematic and polar order which dynamically interconvert in a cyclic fashion (Fig. 4*C*; *SI Appendix*, section 2C; and Movie S4), similar to the numerical results we found for the kinetic Boltzmann equation (Fig. 3*B*). Specifically, nematic bands induce polar wave patterns which propagate along the bands, build up a wave front, and thereby remodel the bands in turn. Furthermore, we observe that polar clusters can remain stable even after penetrating other polar clusters with close to opposite propagation direction (Movie S4).

While the coefficients used in Fig. 4 were chosen mainly for specificity, we found similar results for other choices of coefficients that are consistent with the heuristic requirements discussed above (*SI Appendix*, section 2C and Fig. S8). In fact, we argue that other choices yield similar results as long as the phase diagram features a transition from a regime of nematic bands to polar order for increasing density. Note that this qualitative requirement on the phase diagram is consistent with the experimental observations in actin motility assays (17), where one finds a transition from nematic bands to polar patterns for increasing densities, connected by a regime of interconverting polar and nematic patterns. This qualitative robustness of our results against different parameter choices indicates a more general validity of our results which may also apply to hydrodynamic theories based on other scaling assumptions or truncation schemes.

## Discussion

Motivated by the intriguing dynamic coexistence of polar and nematic patterns observed in recent active-matter experiments and simulations (17), we studied a system of self-propelled particles that exhibit binary nematic alignment interactions with a tunable polar contribution. For a moderate polar bias, our kinetic Boltzmann approach reveals a direct transition from a phase of macroscopic nematic to polar order for high enough densities. In addition to the previously studied nematic bands and traveling waves for respectively small and large polar bias (4, 27), we identify a parameter regime of moderate polar bias and density that exhibits intriguing spatiotemporal dynamics: A dynamic increase of particle density within nematic bands induces a local symmetry-breaking instability and an ensuing transition to polar patterns. This self-organized transition then leads to a rearrangement of the nematic bands which eventually results in a rich set of different final patterns, including coexistence of patterns with nematic and polar symmetry as well as dynamic transformations between them. We find a similar phenomenology in hydrodynamic equations when the coupling between polar and nematic order is strong enough.

Our findings shed additional light on traditional symmetry assumptions in dilute active-matter theories (4) and suggest that the symmetry of patterns can depend on the (nonlinear) dynamics of the system. In the system we studied, a global symmetry-breaking instability of the uniform nematic state first leads to a redistribution of the density into nematic band patterns. Since in our system the density acts as a control (bifurcation) parameter for the macroscopic symmetry, the high-density core of a nematic band can locally cross a threshold value in the density such that there is symmetry breaking; i.e., the symmetry of the system changes from nematic to polar. These local symmetry-breaking transitions eventually lead to the self-organized coexistence of, and cycling between, polar and nematic patterns. In these patterns, nematic and polar patterns are firmly intertwined: Nematic bands serve as scaffolds for the creation of polar wave patterns, which propagate along the nematic band and decay in its low-density neighborhood, which again fuels the formation of nematic bands. All of these observations are in very good agreement with the phenomenology observed in previous actomyosin motility assays (17).

Local steady states and their stability have been found to play an important role in the context of mass-conserving reaction–diffusion systems (40, 41), which have been used to study pattern formation in a broad range of intracellular protein systems. There, following similar principles to those in our study, the local protein densities act as dynamic control variables that determine the local steady states. Since density is diffusively redistributed, this can have particularly dramatic consequences when local steady states become unstable, driving the protein concentrations away from them (40, 42). It will be interesting to further explore the analogies between these nonequilibrium chemical systems, where detailed balance is broken at the level of the chemical reactions, and active systems with (self-)propelled particles. We believe that the observed feedback between pattern formation and local instabilities of steady states is not limited to our study, but could be a more general principle whenever a control parameter (such as density) is dynamically redistributed during pattern formation. From a broader perspective on biological active matter, this could apply whenever individuals dynamically change their microscopic properties (velocity, interaction behavior, etc.) in response to macroscopic parameters such as the density. Prominent examples of such feedback between macroscopic effects and the microscopic components of the system are found in synthetic active systems with chemical interactions (15), collective sensing in bacteria (43–46), and animals (14).

Previous studies on active-matter systems have observed instabilities of nematic band structures in systems with fully nematic alignment interactions between polar particles (28, 47), particles with velocity reversal (48), and apolar particles (49, 50). There, for large enough system sizes, nematic bands exhibit a transverse instability, which causes long undulations and transverse breakup of nematic bands and can lead to chaotic dynamics (28, 47, 49, 50). While our numerical solutions of the hydrodynamics equations also exhibit undulations of nematic bands (Fig. 4 *B* and *C* and Movie S4), our system with mixed alignment symmetry features an additional instability of nematic bands toward polar order parallel to nematic bands, which leads to the formation of polar waves along the bands. We hypothesize that the resulting deformation and remodeling of nematic bands by polar patterns as well as the time and length scales involved in these processes denote an interesting field of research with similarly rich phenomenology and impact to those of transverse band instabilities.

In addition to actomyosin motility assays, patterns with polar and nematic symmetries were commonly suggested in systems of rodlike particles (19–22) and observed in experiments with motile bacteria in liquid crystals (51–53). Moreover, patterns of intertwined symmetries play a prominent role even in equilibrium systems, such as in high-temperature superconductors (54, 55). We believe that it would be promising to further investigate interactions between polar and nematic symmetries in pattern-forming systems with focus on mutual feedback between pattern formation and local symmetry-breaking instabilities (bifurcations) as the cause of dynamic coexistence between patterns of different symmetry.

Our results suggest that the existence of interconverting polar and nematic patterns depends only on qualitative features of the system’s phase diagram (in particular, a transition from a regime of nematic bands to a regime of polar order for increasing density) and not on assumptions on microscopic details. This implies a possible relevance of this phenomenon for a broad class of experimental systems beyond the actomyosin motility assay. We hypothesize that pattern-induced symmetry breaking could serve as a useful and general design principle with broad applications to synthetic and living active matter (51–53) as well as other pattern-forming systems with intertwined symmetries (54, 55).

## Materials and Methods

### Kinetic Approach.

Our kinetic approach is based on a kinetic Boltzmann equation for propelled particle systems first presented in ref. 9. We generalize this approach to account for binary collisions with nematic alignment with a tunable polar bias (for details see *SI Appendix*, section 1). We find approximate uniform stationary solutions of the respective equation system in Fourier space and study their stability against wave-like perturbations following ref. 30. The resulting stability diagram is shown in Fig. 2*A*. The predictions from this diagram are tested in numerical solutions using a modified version of the SNAKE algorithm (29) (for details see *SI Appendix*, section 1B).

### Hydrodynamic Approach.

For our hydrodynamic approach, we study the linear stability as well as numeric solutions of Eq. **4** together with the continuity equation for different coefficients. In the main text and in Fig. 4, we fixed all coefficients except for $\alpha_{2}$ to their values derived explicitly from the kinetic Boltzmann equations for $\bar{\rho }=0.16$, σ=0.2, and ψ=0 (for details see *SI Appendix*, section 2). In addition, we tested different alternative choices that fulfill the conditions discussed in the main text (*SI Appendix*, section 2C and Fig. S8).

## Supplementary Material

Supplementary File

Supplementary File

Supplementary File

Supplementary File

Supplementary File

## Data Availability

All relevant data are within this paper and *SI Appendix*.
