# Supplementary material for: Pattern-induced local symmetry breaking in active-matter systems
Source: Proc Natl Acad Sci U S A. 2020 Nov 30;117(50):31623–30. doi: 10.1073/pnas.2010302117 (PMC7749307; doi:10.1073/pnas.2010302117)
Supplement: Supplementary File [file pnas.2010302117.sapp.pdf]

PNAS
www.pnas.org

Supplementary Information for

Pattern-induced local symmetry breaking in active matter systems

Jonas Denk, Erwin Frey

Erwin Frey.
E-mail: frey@lmu.de

This PDF file includes:

Supplementary text
Figs. S1 to S8
Legends for Movies S1 to S4
SI References

Other supplementary materials for this manuscript include the following:

Movies S1 to S4

Supporting Information Text

1. Kinetic Boltzmann approach

Following Refs. (1, 2), the kinetic Boltzmann equation for the orientational one-particle distribution function $f(\mathbf{r},\theta,t)$ reads:

$$\partial_t f(\mathbf{r},\theta,t) + v_0 \mathbf{e}(\theta)\cdot\partial_{\mathbf{r}} f(\mathbf{r},\theta,t) = \mathcal{I}_{\text{diff}}[f] + \mathcal{I}_{\text{coll}}[f,f]\,, \qquad [1]$$

where $\mathcal{I}_{\text{diff}}$ and $\mathcal{I}_{\text{coll}}$ denote the diffusion and collision integrals, respectively. They are given by

$$\mathcal{I}_d[f] = -\,\lambda f(\theta) + \lambda \int_{-\pi}^{\pi}\mathrm{d}\theta' \int_{-\infty}^{\infty}\mathrm{d}\eta\; f(\theta')\,P_\sigma(\eta)\delta_{2\pi}(\theta'-\theta+\eta)\,, \qquad [2a]$$

$$\mathcal{I}_c[f] = -\,f(\theta)\int_{-\pi}^{\pi}\mathrm{d}\theta'\;\mathcal{R}(\theta,\theta')\,f(\theta') + \int_{-\pi}^{\pi}\mathrm{d}\theta_1\; f(\theta_1)\int_{-\pi}^{\pi}\mathrm{d}\theta_2\;\mathcal{R}(\theta_1,\theta_2)f(\theta_2)\int_{-\infty}^{\infty}\mathrm{d}\eta\; P_\sigma(\eta)\,\Psi_\eta(\theta_1,\theta_2,\theta)\,. \qquad [2b]$$

$P_\sigma(\eta)$ is a Gaussian distribution with standard variation σ and $\delta_{2\pi}$ denotes a generalized Kronecker delta, imposing that the argument is zero modulo 2π. $\mathcal{R}(\theta_1,\theta_2)$ denotes the differential cross section of two particles with orientations θ_1 and θ_2. For disk-like particles with diameter d, $\mathcal{R}(\theta_1,\theta_2)$ is given by $\mathcal{R}(\theta_1,\theta_2) = 4v_0 d\sin(\frac{\theta_1-\theta_2}{2})$ (1, 2). The binary interaction rule enters through the alignment function $\Psi_\eta(\theta_1,\theta_2,\theta)$:

For polar alignment: $\Psi_\eta(\theta_1,\theta_2,\theta) = \delta_{2\pi}\left(\frac{\theta_1-\theta_2}{2} - \theta + \eta\right)$

For antipolar alignment: $\Psi_\eta(\theta_1,\theta_2,\theta) = \frac{1}{2}\delta_{2\pi}\left(\frac{\theta_1-\theta_2}{2} - \theta + \frac{\pi}{2} + \eta\right) + \frac{1}{2}\delta_{2\pi}\left(\frac{\theta_1-\theta_2}{2} - \theta - \frac{\pi}{2} + \eta\right)$

For an interaction rule with variable polar bias ψ we assume polar alignment for an intermediate angle with $|(\theta_1-\theta_2)| < \pi/2+\psi$ and anti-polar alignment otherwise. The parameter $\psi \in [0,\pi/2]$ thus characterizes the strength of the polar bias where for $\psi = 0$ and $\psi = \pi/2$ the collision rule reduces to fully nematic or fully polar collisions, respectively.

In the following, we rescale time, space, and density such that $v_0 = \lambda = d = 1$. Then, the only remaining free parameters are the noise amplitude σ, the polar bias ψ, and the mean particle density $\bar{\rho} = A^{-1}\int_A \mathrm{d}\mathbf{r}\int_{-\pi}^{\pi}\mathrm{d}\theta\, f(\mathbf{r},\theta,t)$ measured in units of $\lambda/(dv_0)$, i.e., the number of particles found within the area traversed by a particle between successive diffusion events. In order to study solutions of the the kinetic Boltzmann equation Eq. (1), it is convenient to expand this equation for f in terms of Fourier modes of the angular variable given by

$$f_k(\mathbf{r},t) = \int_{-\pi}^{\pi}\mathrm{d}\theta\;\mathrm{e}^{\mathrm{i}\theta k} f(\mathbf{r},\theta,t)\,. \qquad [3]$$

The dynamics $f_k(\mathbf{r},t)$ is then given by

$$\partial_t f_k + \frac{v_0}{2}\left[\partial_x(f_{k+1}+f_{k-1}) - \mathrm{i}\partial_y(f_{k+1}-f_{k-1})\right] = -\lambda(1-\mathrm{e}^{-(k\sigma^2)/2})f_k + \sum_{n=-\infty}^{\infty}\mathcal{I}_{n,k} f_n f_{k-n}\,, \qquad [4]$$

where the Fourier transform of the collision integral, $\mathcal{I}_{n,k}$, has contributions coming from polar and antipolar alignments depending on the polar bias ψ:

$$\mathcal{I}_{n,k} = \underbrace{\int_{-\frac{\pi}{2}-\psi}^{\frac{\pi}{2}+\psi}\frac{\mathrm{d}\Delta}{2\pi}\mathcal{R}(|\Delta|)\left[P_\sigma\cos(\Delta(n-\frac{k}{2}) - \cos(\Delta n)\right]}_{\rightsquigarrow\text{polar alignment}} + \underbrace{\int_{\frac{\pi}{2}+\psi}^{2\pi-\frac{\pi}{2}-\psi}\frac{\mathrm{d}\Delta}{2\pi}\mathcal{R}(|\Delta|)\left[P_\sigma\cos(\frac{k\pi}{2})\cos(\Delta(n-\frac{k}{2}) - \cos(\Delta n)\right]}_{\rightsquigarrow\text{antipolar alignment}}\,. \qquad [5]$$

Since $\mathcal{I}_{n,0}=0$ for all n, a state with spatially homogeneous density $\bar{\rho}=f_0$ and all higher Fourier modes vanishing is a stationary solution of Eq. (1) (*disordered state*). To linear order, the dynamics of small perturbations δf_k with respect to this uniform state is given by $\partial_t \delta f_k = \mu_k(\bar{\rho},\sigma)\,\delta f_k$, where $\mu_k(\bar{\rho},\sigma)=(\mathcal{I}_{0,k}+\mathcal{I}_{k,k})\bar{\rho} - \lambda(1-\mathrm{e}^{-(k\sigma)^2/2})$. For a nematic collision rule with polar bias, as considered here, only μ_1 and μ_2 can become positive, defining critical densities $\rho_1^c(\sigma,\psi)$ and $\rho_2^c(\sigma,\psi)$ at $\mu_1(\rho_1^c,\sigma,\psi):=0$ and $\mu_2(\rho_2^c,\sigma,\psi):=0$, respectively. The numerical solutions for $\rho_1^c(\sigma,\psi)$ and $\rho_2^c(\sigma,\psi)$ are given by the blue and red solid lines in Fig. 2A and C, respectively. Above the thresholds ($\bar{\rho}>\rho_2^c$ or $\bar{\rho}>\rho_1^c$), the spatially uniform disordered state is unstable and macroscopic order may emerge.

Varying the polar bias, we find that the transition densities $\rho_1^c(\sigma,\psi)$ and $\rho_2^c(\sigma,\psi)$ increase when the polar bias departs from $\psi=\pi/2$ (fully polar system) or $\psi=0$ (fully nematic system), respectively. Furthermore, $\rho_1^c(\sigma,\psi)$ and $\rho_2^c(\sigma,\psi)$ diverge at small and large finite polar bias [see the divergence of $\rho_1^c(\sigma,\psi)$ at $\psi\approx 0.14$ in Fig. 2(A)], respectively. These divergences can be understood based on the observations in earlier work (1, 3) that in the kinetic Boltzmann approach for purely polar (1) or nematic (3) alignment interactions, the critical densities for the onset of polar or nematic order diverge at certain noise values σ_0^p and σ_0^n, respectively. Above these noise values, σ_0^p and σ_0^n, the systems with purely polar or nematic alignment do not show any transition to polar or nematic order for any density. We find that when departing from the values $\psi=\pi/2$ (fully polar system) or $\psi=0$ (fully nematic system), these critical noise values σ_0^p and σ_0^n both monotonically decrease. When varying the polar bias for a chosen σ ($\sigma=0.2$ in our study), σ_0^p and σ_0^n can drop below this σ for certain values of ψ. As a result, the critical density for the onset of polar or nematic order, respectively, diverges at the respective values of ψ. We believe that the divergence of $\rho^c_{\text{nem-pol}}$ for finite polar bias [as observed in Fig. 2(A)] is a direct consequence of this divergence of ρ_1^c.

A. Stationary, spatially uniform solutions and linear stability analysis. In order to find approximate stationary, spatially uniform solutions $\{f_k^{(0)}\}$ of Eq. (4) in regimes where the disordered state is unstable, we followed Ref. (4) and first numerically calculated the uniform steady state solutions for $\{f_k^{(0)}\}$ with $k\le k_c$ of Eq. (4) setting all angular Fourier modes with $k>k_h$ as well as all spatial and temporal derivatives to zero. In Fig. 2(A),(B) we used $k_c=10$; choosing larger k_c only leads to negligible quantitative changes of the stability diagram Fig. 2(A) and the uniform solutions Fig. 2(B). Depending on the global density $\bar{\rho}$ and the polar bias ψ, we find disordered solutions ($f_k^{(0)}=0$ with $k=1,\dots,k_c$), solutions with purely nematic order ($f_{2k}^{(0)}>0$, $f_{2k-1}^{(0)}=0$ with $k=1,\dots,k_c$), and solutions with polar order ($f_{2k}^{(0)}>0$, $f_{2k-1}^{(0)}>0$ with $k=1,\dots,k_c$).

Next, we studied the linear stability of the these solutions by substituting $\rho=\bar{\rho}+\delta\rho$ and $f_k=f_k^{(0)}+\delta f_k$ where $\delta\rho$ and δf_k denote small perturbations. The linearized set of equations of motion for $\delta\rho_{\mathbf{q}}(t)$, $\delta f_{k,\mathbf{q}}(t)$ and $\delta f^*_{k,\mathbf{q}}(t)$ then read

$$\partial_t \delta f_k = -\frac{v_0}{2}(\nabla\delta f_{k-1}+\nabla^*\delta f_{k+1}) + \lambda(\mathrm{e}^{-(k\sigma^2)/2}-1)\delta f_k + \sum_{n=-\infty}^{\infty}(\mathcal{I}_{n,k}+\mathcal{I}_{k-n,k})|f_{k-n}|\delta f_n\,. \qquad [6]$$

In order to probe spatially non-uniform perturbations, we assume wave-like perturbations of the form

$$\delta\rho(\mathbf{r},t)\sim\delta\rho_{\mathbf{q}}\,\mathrm{e}^{\mathrm{i}\mathbf{q}\cdot\mathbf{r}}\,, \qquad \text{and} \qquad \delta f_k(\mathbf{r},t)\sim\delta f_{k,\mathbf{q}}\,\mathrm{e}^{\mathrm{i}\mathbf{q}\cdot\mathbf{r}}\,, \qquad [7]$$

where $\delta\rho_{\mathbf{q}}$ and $\delta f_{k,\mathbf{q}}$ are in general complex amplitudes that are assumed to be small. Periodic boundary conditions in our numerical solution impose $|\mathbf{q}|=n\frac{2\pi}{L}$, $n\epsilon\mathbb{Z}$, where $L=\sqrt{A}$ and A is the area of the (quadratic) system. Substituting Eq. (7) into Eq. Eq. (6), we solved the resulting linear set of equations for the maximal eigenvalue as a function of the wave vector $\mathbf{q}$. The real part of this eigenvalue sets the linear growth rate of the respective perturbation. A solution $\{f_k^{(0)}\}$ is linearly unstable when there is a perturbation with positive growth rate and stable otherwise. Specifically, we probed the stability of spatially uniform solutions against spatial perturbations with wave vector parallel and perpendicular to the order of the uniform solution.

As summarized in Fig. 2(A), we find that for low densities, the only uniform solution is the disordered state and it is linearly stable. For larger densities we find spatially uniform solutions of purely nematic order or polar order that are stable against perturbations for arbitrary $\mathbf{q}$ (denoted respectively as uniform nematic and polar states in Fig. 2(A)). Moreover, we find regimes in which solutions with purely nematic order or polar order are stable against uniform perturbations, i.e. $|\mathbf{q}| = 0$, but not against spatial perturbations, i.e. $|\mathbf{q}| > 0$. For solutions with nematic order, the growth rate of perturbations in the respective regime is maximal when the wave vector of the perturbation is *perpendicular* to the nematic order, while for solutions with polar order, the growth rate of perturbations in the respective regimes is maximal when the wave vector of the perturbation is *parallel* to the polar order. This indicates the formation of nematic band patterns and polar wave patterns, respectively (as shown in Fig. 2(A)).

Bistability regime. Close to the intersection of the phase boundaries ρ_1^c and ρ_2^c, there is a parameter regime (hatched area in Fig. 2(A) where we find bistability in the following sense: In this regime, linear stability analysis shows that both a uniform disordered state and a polar ordered state are linearly stable against spatially uniform perturbations. However, linear stability analysis also shows that the spatially uniform polar ordered state is unstable against spatial perturbations. This indicates that both the disordered state and polar wave patterns represent metastable states and can be attractors of the dynamics depending on the initial conditions. In our numerical solutions shown in for Fig. 2(C) and Fig. 3, (see Sec. 1B for details on the algorithm) we choose disordered initial conditions and therefore only observe the disordered state in the regime of bistability. In fact, when initializing our numerical solution with a polar wave state, which we obtained by numerically solving the kinetic Boltzmann equation for parameters in the regime of polar patterns, we find polar wave patterns also in the bistability regime. In a more detailed analysis of this regime it would be interesting to test different initial conditions including spatially non-uniform or polar ordered initial conditions. Given the metastability of the disordered solution, one could ask about the size of a 'critical nucleus', that potentially triggers a transition from a disordered to a polar wave state, and study many more interesting questions concerning the ensuing coarsening process (5).

Within the range of polar bias values that limit the regime of bistability [approximately between $\psi/\pi = 0.1$ and $\psi/\pi = 0.15$ in Fig. 2(A)], the transition line marked by $\rho_1^c(\psi)$ can be understood as the "spinodal", i.e. for densities above ρ_1^c, polar patterns form spontaneously. In the same sense, the line demarcating the bistable region can be considered as a "binodal", i.e. for densities above this line (but below ρ_1^c) polar patterns are metastable and require a large enough perturbation of the disordered state to be able to form. In this range of the polar bias the mean-field solution (i.e. the spatial uniform solution $f_k^{(0)}$) shows a subcritical bifurcation (see Fig. S1, $\psi/\pi = 0.113$ and $\psi/\pi = 0.138$). Discontinuous transitions in the spatially uniform solutions have been suggested previously in agent-based simulations for a system of self-propelled aligning hard discs (6, 7). The bistable regime is bounded by the intersection points of the line $\rho^c_{\text{nem-pol}}(\psi)$ with the line $\rho_2^c(\psi)$ (at $\psi/\pi \approx 0.1$) and the intersection point of the curve that demarcates bistable regime and the line $\rho_1^c(\psi)$ (at $\psi/\pi \approx 0.15$), which are both cusp bifurcations of the mean-field theory. For larger polar bias, $\psi/\pi \gtrapprox 0.15$ (Fig. S1, $\psi/\pi = 0.178$), mean-field theory shows the well-studied supercritical bifurcation as observed in (mean-field) polar active matter (1, 2).

B. Numerical solutions. In order to study the nonlinear dynamics and steady states in the kinetic Boltzmann equation in real space Eq. (1) we employed the `SNAKE` algorithm as introduced in Ref. (8). This algorithm is based on finite-difference method on a regular lattice with discrete time steps and convenient tessellations of the one-particle distribution function $f(\mathbf{r}, \theta, t)$ into angular slices (each slice corresponds to a certain angular range). At each discrete time step, the one particle distribution on every lattice site is updated according to the different terms in the kinetic Boltzmann equation Eq. (1) that account for convection between lattice sites as well as diffusion and binary collisions (density exchange between angular slices). As discretization we used a quadratic periodic regular lattice with periodic boundary conditions with equally sized angular slices. The number of angular slices varied from 40 to 80. To account for an alignment rule between the angular slices θ_i and θ_j with variable polar bias we assume polar alignment for an intermediate

angle $|(\theta_i - \theta_j)| < \pi/2 + \psi$ and antipolar alignment otherwise. For Figs. 2(B,C), we used only one lattice point in order to obtain the spatially uniform solution, while for Fig. 3 we used a 60×60 lattice with lattice spacing 10. Hence, the simulated system size is $A = 600 \times 600$. The computation time step was set to 0.3. The system was initialized with a disordered state with small random density fluctuations around the mean density $\bar{\rho} = A^{-1} \int_A \rho(\mathbf{r}, t)$. For densities $\bar{\rho}$ close to the onset of order we find the formation of spatial patterns: in the regime where our linear stability analysis [Fig. 2(A)] predicts polar patterns we observe traveling wave patterns as reported in Refs. (8) for fully polar alignment [see Fig. S2(A)]. For vanishing and small polar bias and close to ρ_2^c we see the formation of nematic band patterns [see Fig. S2(B)]. The regimes of patterns and uniformly ordered solutions are in good agreement with our linear stability analysis. Similar to previous comparisons between linear stability analysis and solutions based on the `SNAKE` algorithm(4), the regimes of patterns in our numerical solutions are more restricted in parameter space than predicted by linear stability analysis [Fig. 3(A)], probably due to numerical diffusion in the discrete implementation of the `SNAKE` algorithm (9).

As detailed in the main text, for intermediate polar bias and densities, we find initially forming nematic bands which undergo an instability of polar order and eventually transform into patterns of coexisting and cycling polar and nematic symmetry (see Movie S1). We argue that this instability occurs since the density in the high density core of the nematic band exceeds the transition density $\rho^c_{\text{nem-pol}}$, above which polar order grows exponentially. This is shown in Fig. S3, where we plot the local dynamics of the density, the polar and nematic order parameter within a nematic band and in the disordered area between two bands.

Depending on the system size and random seed of initial conditions, we observe that the polar instability within nematic bands can lead to different patterns with polar and nematic symmetry including replacement of nematic bands by polar waves [see Movie S2], coexistence of nematic bands and polar waves [see Movie S1] or alternating formation of nematic bands and polar waves [see Movie S3]. The observation of different states, depending on the initial conditions, suggests that these states are metastable. It will be interesting to explore the stability as well as possible transitions between these states in the presence of spatial or temporal perturbation.

Transitions between polar and nematic regimes. Earlier work on the kinetic Boltzmann equation for systems with purely polar or nematic alignment symmetries suggest that, while the spatially uniform solutions (mean-field solutions) of the respectively polar or nematic order parameter undergo a continuous transition at their onset, spatial fluctuations lead to a discontinuous transition to respectively polar or nematic patterns (10). Similarly, while the uniform solutions of the kinetic Boltzmann equation show a continuous transitions [see Fig. 2(B)], the numerical solutions of a spatially extended system suggest that the nematic and the polar order both undergo a discontinuous transition at their onset densities. In more detail, the amplitude of the nematic order parameter, $|f_2|$, jumps from zero to a finite value at ρ_2^c [see Fig. S4]. Similarly, the amplitude of the polar order parameter, $|f_1|$, jumps from zero to a finite value at a certain threshold density, which depends on the polar bias: For small polar bias, we find that for increasing $\bar{\rho}$ there is first a transition from a regime of nematic band patterns to uniform solutions with nematic order before at $\rho^c_{\text{nem-pol}}$, polar order jumps to a finite value [see Fig. S4(A), compare also Fig. 3(A)]. For larger polar bias we find that for increasing density, $|f_1|$ already jumps at a density value smaller than $\rho^c_{\text{nem-pol}}$, which marks the regime of 'nematic-polar patterns' in Fig. 3(A) [see Fig. S4(B)]. At each transition, the spatial variance of the density, var(ρ), is nonzero, indicating the presence of patterns. It will be interesting to further investigate the transitions between nematic and polar states and the possible metastability in their vicinity more closely in follow-up studies. For instance, similar as in the bistability regime discussed above, it would be interesting to test different initial conditions including spatially non-uniform or polar ordered initial conditions. Given the metastability of the disordered solution, one could ask about the size of a 'critical nucleus', that potentially triggers transitions between states with nematic order and polar wave state, and study many more interesting questions concerning the ensuing coarsening process (5). Previous studies based on hydrodynamic equations for active matter with polar symmetry (11) suggest that in the limit of infinite system size the transition from disorder to polar patterns becomes continuous. Likewise, it

would be interesting to investigate the transitions of nematic and polar order in our system for increasing system sizes in order to study their properties in the thermodynamic limit.

2. Hydrodynamic approach

Complementary to kinetic approaches, hydrodynamic theories have served as a useful basis to study active matter systems (12). These theories are either based on symmetry assumptions or derived directly from kinetic theories at the onset of macroscopic order. In Section A below, we explicitly discuss a derivation of hydrodynamic equations for our system with a polar bias, using scaling assumptions that were previously employed for a system with purely nematic alignment (3). In Section B, we argue that modes that have been neglected in this approach might be relevant for an explicit derivation of the hydrodynamic coefficients. In Section C, we explore the hydrodynamic equations for general coefficients and find a similar phenomenology as in our kinetic approach.

A. Derivation of hydrodynamic equations from the kinetic Boltzmann approach. In order to derive closed hydrodynamic equations from the kinetic Boltzmann equation Eq. (1), we follow Ref. (13) and assume that close to the onset of polar or nematic order the respective fields f_1 and f_2 as well as temporal and spatial variations are small. This assumption suggests scaling relations which allow to truncate the infinite sum in Eq. (4) such that one obtains a closed set of equations for the dominant hydrodynamic fields.

Balancing terms in the Boltzmann equation Peshkov et al. (3) have proposed scaling relations for a system of polar particles with fully nematic collisions (i.e. $\psi = 0$) according to

$$\rho - \bar{\rho} \sim \varepsilon,\ \{f_{2k-1}, f_{2k}\}_{k\geq 1} \sim \varepsilon^k,\ \partial_t \sim \varepsilon,\ \partial_{x/y} \sim \varepsilon\,. \qquad [8]$$

With these scaling relations, one can expand the sum in Eq. (4) retaining only terms of order ε^3 to get closed equations for the order fields $f_{1,2,3,4}$. The equations for f_3 and f_4 yield expressions for f_3 and f_4 in terms of f_1 and f_2 and one arrives at the following hydrodynamic equations for ρ, f_1, and f_2:

$$\partial_t \rho = -\frac{1}{2}(\nabla f_1^* + \nabla^* f_1)\,, \qquad [9a]$$

$$\partial_t f_1 = -(\alpha_0 + \rho\alpha_1) f_1 + \alpha_2 f_1^* f_2 - \alpha_3 |f_2|^2 f_1 - \frac{1}{2}(\nabla\rho + \nabla^* f_2) + \gamma_1 f_2^* \nabla f_2\,, \qquad [9b]$$

$$\partial_t f_2 = (-\beta_0 + \rho\beta_1) f_2 + \beta_2 f_1^2 - \beta_3 |f_2|^2 f_2 - \beta_3' |f_1|^2 f_2 - \frac{1}{2}\nabla f_1 + \gamma_2 \nabla\nabla^* f_2 - \gamma_3 f_1^* \nabla f_2 - \gamma_4 \nabla^*(f_1 f_2)\,, \qquad [9c]$$

where $\nabla := \partial_x + \mathrm{i}\partial_y$ and the star denotes complex conjugation. The coefficients are given by

$$\alpha_0 = 1 - P_1(\sigma)\,, \qquad [10a]$$
$$\alpha_1 = -\left(\mathcal{I}_{0,1}(\sigma) + \mathcal{I}_{1,1}(\sigma)\right)\,, \qquad [10b]$$
$$\alpha_2 = \mathcal{I}_{-1,1}(\sigma) + \mathcal{I}_{2,1}(\sigma)\,, \qquad [10c]$$
$$\alpha_3 = -4\gamma_2 \left(\mathcal{I}_{-2,1}(\sigma) + \mathcal{I}_{3,1}(\sigma)\right)\left(\mathcal{I}_{1,3}(\sigma) + \mathcal{I}_{2,3}(\sigma)\right)\,, \qquad [10d]$$
$$\beta_0 = 1 - P_2(\sigma)\,, \qquad [10e]$$
$$\beta_1 = \mathcal{I}_{0,2}(\sigma) + \mathcal{I}_{2,2}(\sigma)\,, \qquad [10f]$$
$$\beta_2 = \mathcal{I}_{1,2}(\sigma)\,, \qquad [10g]$$
$$\beta_3 = -\mathcal{I}_{2,4}(\sigma)\left(\mathcal{I}_{-2,2}(\sigma) + \mathcal{I}_{4,2}(\sigma)\right) / \left(\left(1 - P_4(\sigma) - \bar{\rho}\left(\mathcal{I}_{0,4}(\sigma) + \mathcal{I}_{4,4}(\sigma)\right)\right)\right)\,, \qquad [10h]$$
$$\beta_3' = -4\gamma_2 \left(\mathcal{I}_{1,3}(\sigma) + \mathcal{I}_{2,3}(\sigma)\right)\left(\mathcal{I}_{-1,2}(\sigma) + \mathcal{I}_{3,2}(\sigma)\right)\,, \qquad [10i]$$
$$\gamma_1 = -2\gamma_2 \left(\mathcal{I}_{3,1}(\sigma) + \mathcal{I}_{-2,1}(\sigma)\right)\,, \qquad [10j]$$
$$\gamma_2 = 1/\left(4\left(1 - P_3(\sigma) - \bar{\rho}\left(\mathcal{I}_{3,3}(\sigma) + \mathcal{I}_{0,3}(\sigma)\right)\right)\right)\,, \qquad [10k]$$
$$\gamma_3 = 2\gamma_2 \left(\mathcal{I}_{-1,2}(\sigma) + \mathcal{I}_{3,2}(\sigma)\right)\,, \qquad [10l]$$
$$\gamma_4 = 2\gamma_2 \left(\mathcal{I}_{1,3}(\sigma) + \mathcal{I}_{2,3}(\sigma)\right)\,, \qquad [10m]$$

where $P_k(\sigma) = e^{-(k\sigma)^2/2}$ and $\mathcal{I}_{n,k}(\sigma)$ are collision integrals defined in Eq. (5). For a fully nematic collision rule, the coefficients α_0, α_1, α_2, α_3, β_0, β_1, β_2, β_3 are positive. As discussed in the main text, this defines a critical density $\bar{\rho} = \beta_0/\beta_1$ above which the disordered state is unstable against growth of nematic order, whereas polar order will always decay to linear order. In principle, one could argue that these equations, which were derived for a fully nematic collision rule, might still be useful to study a system including a small polar bias. Indeed, the coefficient α_1 becomes negative for larger polar bias defining a critical density at $\bar{\rho} = -\alpha_0/\alpha_1$ above which the disordered state is linearly unstable against growth of polar order. The transition densities for nematic order $\bar{\rho} = -\beta_0/\beta_1$ and polar order $\bar{\rho} = -a_0/a_1$ are in fact equivalent representations of the conditions $\mu_2(\bar{\rho}, \sigma, \psi) = 0$ and $\mu_1(\bar{\rho}, \sigma, \psi) = 0$, respectively, derived in the main text. We therefore included the dependence of the collision integrals on the polar bias ψ as given by Eq. (5). Even for $(\alpha_0 + \alpha_1\rho) > 0$ equation Eq. (9b) might allow a polar instability when the second term, which is linear in f_2, dominates the first term and leads to an overall positive growth rate of f_1.

Mean-field phase diagram. Next we tested the validity of the hydrodynamic equations for systems with a polar bias, Eq. (9), derived using the closure scheme adapted from the analysis of purely nematic systems (3). To this end, we performed a numerical linear stability analysis of the hydrodynamic equations at the mean-field level, i.e. assuming spatially uniform solutions. The ensuing phase diagram is shown in Fig. S5 for a fixed noise value of $\sigma = 0.2$; this value is only chosen for specificity.

At high densities, it exhibits a transition from nematic to polar order already for vanishing and small polar bias. This unphysical feature is likely an artefact of the truncation procedure which is well-suited for densities close to the order transition only. Previous numerical analysis for systems with purely nematic collision rules have observed unbounded growth (3) for high densities, indicating that there higher orders neglected in the derivation of Eq. (9) become important (3). The mean-field phase diagram also features a regime of polar order for larger polar bias bias. Here, similar as in Fig. 2(C), polar order is not restricted to densities above the threshold to polar order, ρ_1^c, but can also emerge in a regime above the transition to nematic order marked by ρ_2^c. However, unlike the phase diagram for the kinetic Boltzmann equation [Fig. 2(C)], the phase diagram Fig. S5 of the hydrodynamic theory, Eq. (9), lacks a pronounced transition from a phase with nematic to a phase with polar order upon increasing the density. The transition line $\rho_{\text{nem-pol}}^{(c,h)}$ between the nematic and polar phase (dashed line in Fig. S5) show qualitatively different behavior than $\rho_{\text{nem-pol}}^{c}$ [dashed line in Fig. 2(C)] as a function of density and polar bias. Taken together, the hydrodynamic theory Eq. (9) with the coefficients given by Eq. (10) shows a mean-field phase diagram that is qualitatively different from the phase diagram obtained with the kinetic Boltzmann equation.

Critical assessment of closure schemes. The failure of the closure scheme in Ref. (3) for systems with polar bias made us wonder whether other closure schemes could possibly give the qualitatively correct mean-field behavior. Close to the nematic to polar transition the assumed similar scaling of polar and nematic order in Ref. (3) seems indeed not fully plausible. For this reason, among others, we have tested a variety of other closures schemes including schemes similar to the ones discussed in Ref. (14). To list just a few, we tested closure schemes which take into account higher orders in ϵ, use different scaling assumptions for odd and even angular Fourier modes, and schemes where saturating terms of higher orders in the gradients are included. Moreover, close to the nematic-polar transition numerical solutions of the kinetic Boltzmann equation show that higher even modes (e.g. the fourth and the sixth angular Fourier modes, f_4 and f_6) assume values much higher than the polar order, suggesting closures that may even take into account the explicit dynamics of these higher even modes. Accordingly, we tested closure schemes that yield closed dynamic equations not only for the density, the polar and the nematic order, but also for higher orders.

However, when using the coefficients explicitly derived from the kinetic Boltzmann equation and the respective closure scheme, none of the derived sets of hydrodynamic equations was able to reproduce the results obtained from the full kinetic Boltzmann equation, not even qualitatively. Either, the linear stability analysis did not show a transition from a polar to a nematic phase for increasing density, and, as a result, numerical solutions did not show a pattern-induced symmetry breaking with intertwined polar and nematic

patterns (similar to the phase diagram in Fig. S5). Or, the linear stability analysis suggested a transition from a nematic to a polar phase for increasing density, but the numerical solutions (see Sec. 2B for details) showed intertwined nematic and polar patterns that quickly diverged (similar to the numerical solutions discussed along Fig. S6).

Based on these extensive analyses, we concluded that it is very difficult to find a closure scheme of the kinetic Boltzmann equation that explicitly yields coefficients that correctly account for the scaling of polar and nematic orders at both, the disorder-nematic order transition and the nematic-polar order transition. Of course, we do not want to – and cannot – fully exclude the possibility of finding a heuristic closure scheme that yields coefficients that reproduces our results. However, for our study we decided to employ hydrodynamic equations that are simple enough to explain pattern-induced symmetry breaking in a comprehensible way and whose constituting terms are familiar and well-founded through various active matter approaches including Smoluchowski, Fokker-Planck and kinetic Boltzmann approaches. In return, we do not fix the coefficients as explicit functions of the density and noise value through the kinetic Boltzmann equation. From a technical point, it may be attractive to derive the coefficients explicitly as functions of density and noise (as possible for the fully nematic system, Ref. (3)). However, we think that our finding that patter-induced symmetry breaking can occur when the phase diagram features a transition from a nematic band phase to polar order for increasing density strongly suggests that patter-induced symmetry breaking is more general for active matter theories and – at least to some extend – independent of the underlying scaling assumptions.

B. Numerical solutions including higher angular Fourier modes. The difference between the mean-field phase diagrams shown in Fig. S5 and in Fig. 2(C) suggests that higher order modes, which have been neglected in the derivation of Eq. (9), must be accounted for in order to correctly capture the transition between nematic and polar patterns. To test this conjecture, we numerically solve the Boltzmann equation in angular Fourier space Eq. (4) with the collision integrals defined in Eq. (5) taking into account modes f_k with $|k|$ up to a certain k_h and setting all modes with $|k| > k_h$ and their derivatives to zero. Furthermore, we assumed periodic boundary conditions. For the numerical solution we used `XMDS2` (15), a spectral solver based on fast Fourier transformation (FFT). In the parameter regime, where the numerical solutions of the Boltzmann equation in real space [see Section 1B above] show transitions between nematic bands and polar patterns, we find the formation of nematic bands which undergo a polar instability [see Fig. S6]. Moreover, this polar instability does not lead to saturation in the polar order but actually to a divergence in the polar order parameter.

C. Generalized hydrodynamic equations. In our mansucript we take a *semi-phenomenological* approach which retains the structure of Eq. (9) but investigates the dynamics of this field theory for general coupling parameters. For specificity, for our linear stability analysis and numerical solutions of the hydrodynamic

equations Eq. (4) shown in Fig. 4 we use the parameters values

$$\alpha_0 = 0.019801326693244747\,, \quad [11a]$$
$$\alpha_1 = 0.4496250137624467\,, \quad [11b]$$
$$\alpha_3 = 2.1897054378862726\,, \quad [11c]$$
$$\beta_0 = 0.07688365361336424\,, \quad [11d]$$
$$\beta_1 = 0.48867097895034317\,, \quad [11e]$$
$$\beta_3 = 3.007210976282877\,, \quad [11f]$$
$$\beta_2 = -0.12486399707430346\,, \quad [11g]$$
$$\beta_3' = -0.612545745821566\,, \quad [11h]$$
$$\gamma_1 = 0.5258429516101398\,, \quad [11i]$$
$$\gamma_2 = 0.44601012183492805\,, \quad [11j]$$
$$\gamma_3 = 0.14709871812254954\,, \quad [11k]$$
$$\gamma_4 = 1.857267053107399\,, \quad [11l]$$

where units have been suppressed but can be easily read of from Eq. (9). This corresponds to the values in Eq. (10) for $\sigma = 0.2$ and a density $\bar{\rho} = 0.16$ close above the critical density ρ_2^c. As discussed in the main text, we now vary the nematic-polar coupling strength α_2 and $\bar{\rho}$. The stability diagram shown in Fig. 4(A) was obtained by calculating the stationary uniform solutions of Eqs. Eq. (9) and their linear stability against spatially uniform perturbations and non-uniform, wave-like perturbations. Using `XMDS2` (15) and a system size of 200 on a 80×80 lattice, we scanned the parameter regime as indicated in Fig. 4(B) and found regimes of stable nematic band patterns, polar wave patterns, and transformations between nematic patterns due after local instabilities in the high density cores of nematic bands. Fig. S7 shows the local density and nematic order which cross the critical nematic order above which polar order grows. In the parameter regime of transformations between nematic and polar patterns, for large system sizes we observe intriguing patterns of coexisting polar waves and nematic bands which closely interact and transform into each other in a cyclic fashion [Fig. 4(D), Movie S4], similarly as observed in Ref. (16). Our results suggest that the observation of pattern-induced symmetry breaking relies on the topology of the stability diagram, and in particular on the existence of a transition between a regime of nematic patterns and a regime of polar order. Indeed, testing coefficients other than in Eq. (11) we find a similar phenomenology, including patterns with intertwined polar and nematic symmetries for different choices of coefficients, when the respective stability diagram features a transition between nematic band states and polar order. To give an example, Fig. S8(A) (background colors) shows the order of spatially uniform solutions of Eq. (9) and indicates their linear

stability against spatially non-uniform, wave-like perturbations, for the coefficients

$$\alpha_0 = 0.011\,, \tag{12a}$$
$$\alpha_1 = 0.44\,, \tag{12b}$$
$$\alpha_3 = 4.4\,, \tag{12c}$$
$$\beta_0 = 0.044\,, \tag{12d}$$
$$\beta_1 = 0.59\,, \tag{12e}$$
$$\beta_3 = 6.6\,, \tag{12f}$$
$$\beta_2 = -0.16\,, \tag{12g}$$
$$\beta_3' = -1.0\,, \tag{12h}$$
$$\gamma_1 = 1.0\,, \tag{12i}$$
$$\gamma_2 = 0.85\,, \tag{12j}$$
$$\gamma_3 = 0.23\,, \tag{12k}$$
$$\gamma_4 = 3.7\,. \tag{12l}$$

where units have been suppressed but can be easily read of from Eq. (9). These coefficients correspond to the values in Eq. (10) for $\sigma = 0.15$ and a density $\bar{\rho} = 0.08$ close above the respective critical density ρ_2^c, rounded up to two significant digits. As in Fig. 4(A), we varied the nematic-polar coupling strength α_2 and $\bar{\rho}$. Importantly, our linear stability analysis [background colors in Fig. S8(A)] exhibits a transition, marked by $\rho^{(c,h)}_{\text{nem-pol}}$, between a phase of nematic band patterns (yellow background) and polar waves (green background) for increasing density. The symbols in Fig. S8(A) display numerical solutions of Eq. (9) using `XMDS` (15) software with the coefficients Eq. (12) and a system size of 200 on a 80×80 lattice. The numerical solutions show good agreement with the predictions from the linear stability analysis. Furthermore, they show a regime of patterns with intertwined polar and nematic orders (nematic-polar patterns), similar as for the coefficients Eq. (11) [Fig.4(A)]. This supports the generality of our results. In particular, it suggests that the observation of pattern-induced symmetry breaking does not rely on the underlying scaling assumptions that yield explicit expressions for the coefficients, but occurs more generally in systems that feature a direct transition between nematic band regimes and regimes of polar order.

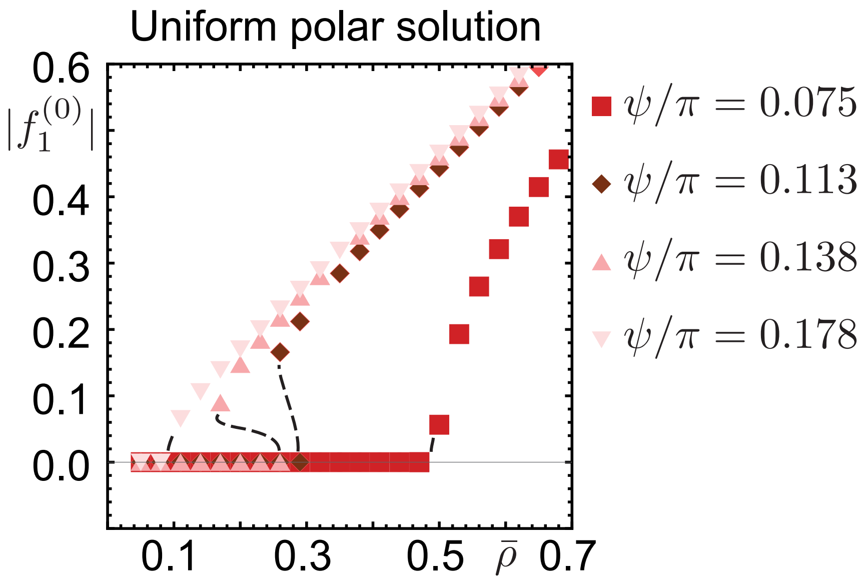

Fig. S1. Subcritical bifurcation of the uniform solutions within the bistability regime. Within the range of polar bias values that limit the regime of bistability [approximately between $\psi/\pi = 0.1$ and $\psi/\pi = 0.15$ in Fig. 2(A)] the uniform solution of the polar order, $|f_1^{(0)}|$ (see Sec. 1A), shows a subcritical transition. For smaller and larger polar bias, the bifurcation is supercritical (continuous in $|f_1^{(0)}|$). The black dashed lines are added to highlight the super- and subcritical shapes of the bifurcation for $|f_1^{(0)}|$ within and out of the range of polar bias that limit the regime of bistability, respectively. Symbols denote numerical solutions for different polar bias (for details on how we obtained the spatially uniform states see Sec. 1A, $k_c = 10$).

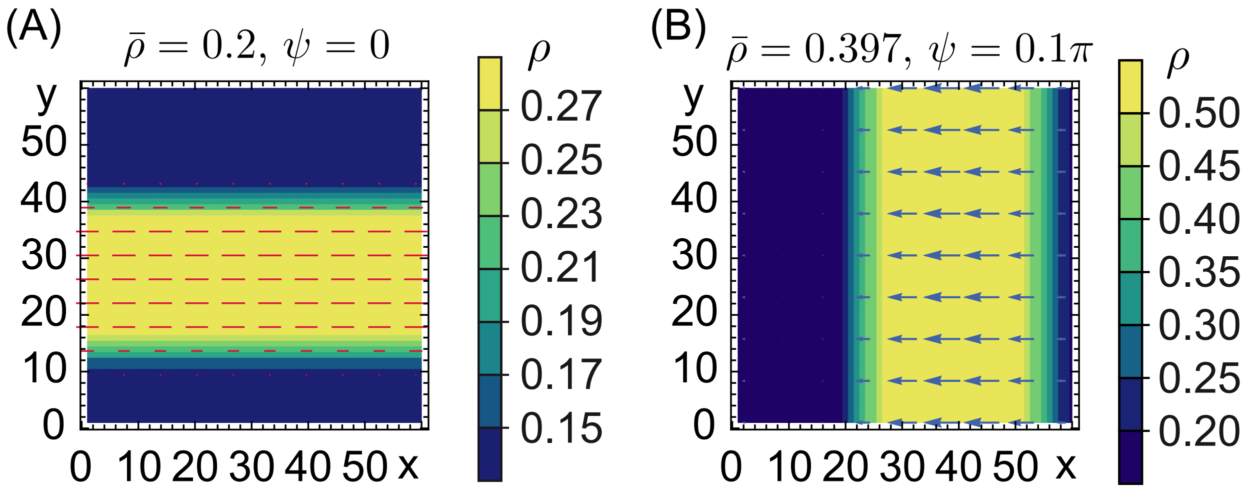

Fig. S2. Spatial patterns. Solving the Boltzmann equation numerically using the SNAKE algorithm (8) (see Section B) we find—for different values of $\bar{\rho}$ and polar bias ψ indicated in the graph—nematic bands (A) and polar traveling waves (B), in agreement with linear stability analysis shown in Fig. 2(A). The local density is given in terms of a color scheme indicted in the graph, the orientation and strength of local nematic and polar order are indicated by red bars and blue arrows, respectively. The numerical solutions used a 60×60 lattice with a spacing of 10 and 80 angular slices. The time step was set to 0.3. Space (x and y) is given in units of the lattice spacing.

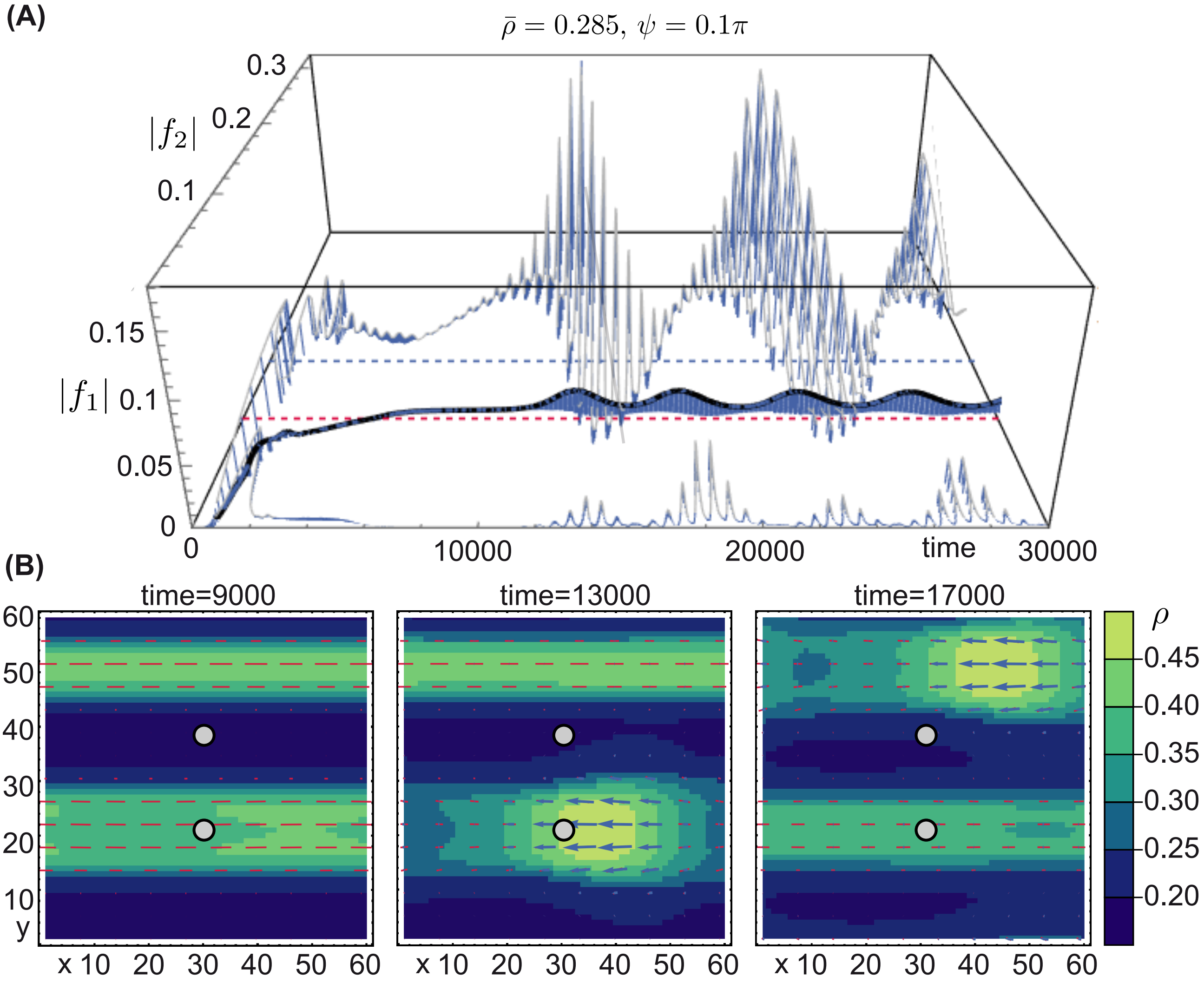

Fig. S3. Polar instability in the kinetic Boltzmann approach. The figure shows the degree of local and average polar and nematic order for patterns with intertwined polar and nematic order at $\bar{\rho} = 0.285$ and $\psi/\pi = 0.1$ (as in Fig. 3(B) and Movie S1). In the numerical solution we used a 60×60 lattice with a spacing of 10 and 80 angular slices. The time step was set to 0.3. As initial condition we used a disordered state with small non-uniform fluctuations (see Section 1B for details on the numerical solution). **(A)** Time trace of nematic and polar order parameters (respectively $|f_2|$ and $|f_1|$) close to the core of a nematic band and in the disordered region of nematic band pattern (gray lines) as well as the average nematic and polar order (black line). The value of the polar amplitude $|f_1|$ along the time traces is indicated by blue bars to clearly visualize the growth of polar order. The upper gray line represents the time trace inside the nematic band (at the location indicated by the lower grey circles in (B) at $(x, y) = (30, 20)$) and the lower gray line represents the time trace in the adjacent disordered region (at the location indicated by the upper grey circles in (B) at $(x, y) = (30, 40)$). The red dashed line indicates the nematic order of the uniform solution. The blue dashed line indicates the nematic order of the uniform solution at the critical density $\rho^c_{\text{nem-pol}}$ (for details on how we calculated the uniform solutions, see Section 1A). After the formation of a nematic band, the nematic order within the band (upper grey line) eventually exceeds the value corresponding to $\rho^c_{\text{nem-pol}}$ while in the disorder region the nematic order drops to zero (lower gray line). After some time polar order within the band (vertical component of the upper grey line) and average polar order (vertical component of the black line) start to grow. **(B)** Snapshots at different time points before and after the polar instability shown in (A). For the respective movie, see Movie S1. The local density is given in terms of a color scheme indicated in the graph, the orientation and strength of local nematic and polar order are indicated by red bars and blue arrows, respectively. Space (x and y) is given in units of the lattice spacing. The gray circles indicate the locations where the time traces in (A) were taken.

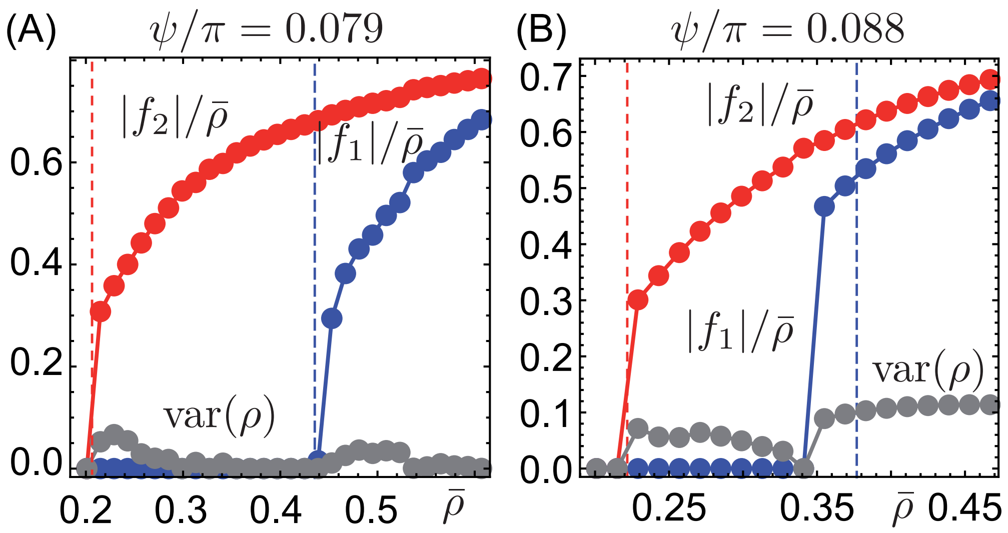

Fig. S4. Transitions between regimes of nematic and polar order. Red, blue and grey dots indicate the average nematic order, average polar order, and the density variation, respectively, in our numerical solution of the kinetic Boltzmann equation Eq. (1) based on the SNAKE (8) algorithm (see 1B). The vertical dashed red and blue lines indicate the transition densities ρ_2^c and $\rho^c_{\text{nem-pol}}$, respectively, as shown in Fig. 2A. **(A)** For small polar bias ($\psi/\pi - 0.079$), our numerical solutions show a jump in the average nematic order and the density variation from zero to nonzero values at ρ_2^c, indicating a discontinuous transition between disorder and nematic patterns. Similarly, at $\rho^c_{\text{nem-pol}}$, our solutions show a jump in the polar order and the density variation from zero to nonzero values, indicating a discontinuous transition from uniform nematic order (without polar order) to patterns with polar order. **(B)** For larger polar bias ($\psi/\pi - 0.088$), our solutions show a discontinuous transition from zero to nonzero polar order already below $\rho^c_{\text{nem-pol}}$, in line with our observation of patterns with intertwined polar and nematic order already below $\rho^c_{\text{nem-pol}}$ [Fig. 3(A), 'nematic-polar patterns' regime].

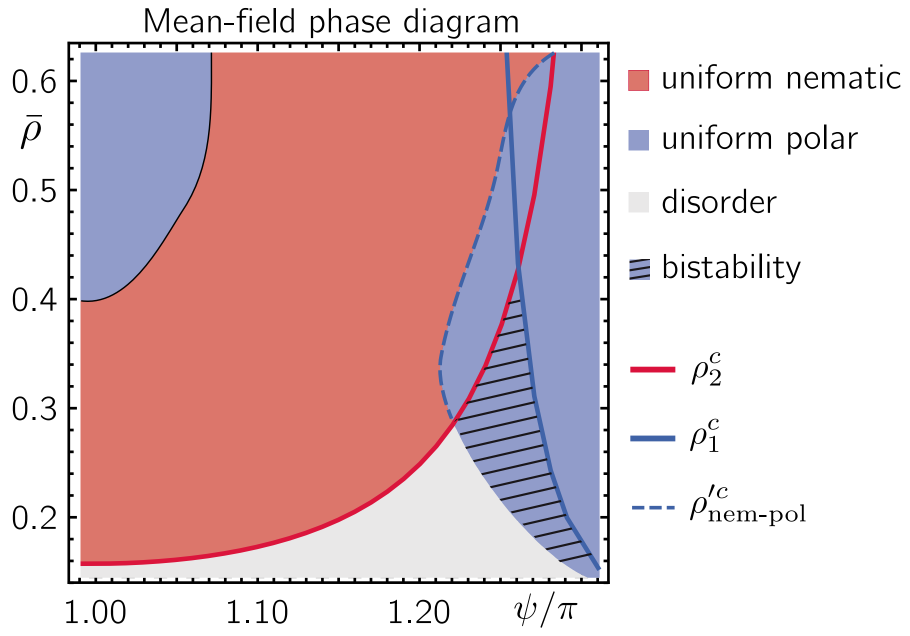

Fig. S5. Mean-field phase diagram of the hydrodynamic equations obtained from the Boltzmann equation using the closure relation from Ref. (3) Colors indicate the type of order of the linearly stable uniform solutions for the hydrodynamic equations Eq. (9) in a spatially uniform system. Above the red solid line (ρ_2^c) and blue solid line (ρ_1^c), the disordered solution is linearly unstable against small spatially uniform perturbations in the nematic and polar order, respectively. The dashed blue line demarcates a regime with linearly stable solutions with polar order. In the hatched regime we observe bistability, i.e. both the disordered solution and a solution with nonzero polar order are linearly stable.

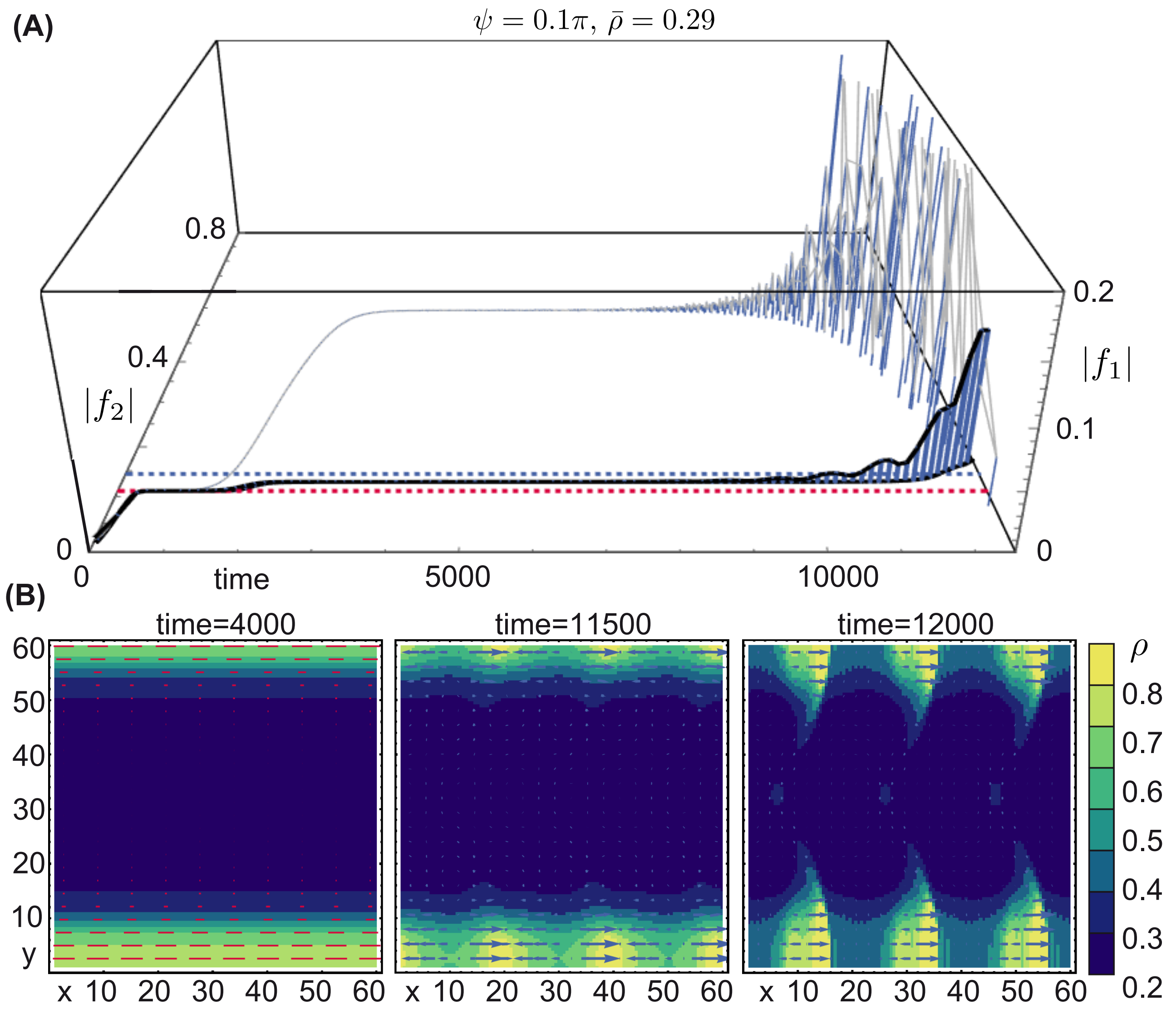

Fig. S6. Polar instability in the kinetic Boltzmann equation in Fourier space. The figure shows the local and average polar and nematic order for the dynamics given by the Eq. (4) with the collision integrals defined in Eq. (5) at $\bar{\rho} = 0.29$ and $\psi/\pi = 0.1$. The solutions were obtained using XMDS software (15) and using a cutoff $k_h = 12$ as detailed in Section B. As initial condition we used a disordered state with small non-uniform fluctuations. **(A)** Evolution of the nematic and polar order of one position in the core of a nematic band (grey line) and of the spatial average (black line). The red dashed line indicates the nematic order of the uniform solution. The blue dashed line indicates the nematic order of the uniform solution at the critical density $\rho^c_{\text{nem-pol}}$ (for details on how we calculated the uniform solutions, see Section 1A). The value of the polar amplitude $|f_1|$ along the time trace is indicated by blue bars to clearly visualize the growth of polar order. Subsequent to the formation of a nematic band, the nematic order within the band (grey line) eventually exceeds the value corresponding to $\rho^c_{\text{nem-pol}}$. After some time polar order within the band (vertical component of the grey line) and average polar order (vertical component of the black line) start to grow. Soon after the polar order grows the numerical solution diverges. **(B)** Snapshots of the patterns at different time points. The color denotes the local density. Red bars and blue arrows indicate the orientations and strengths of the local nematic and polar order, respectively.

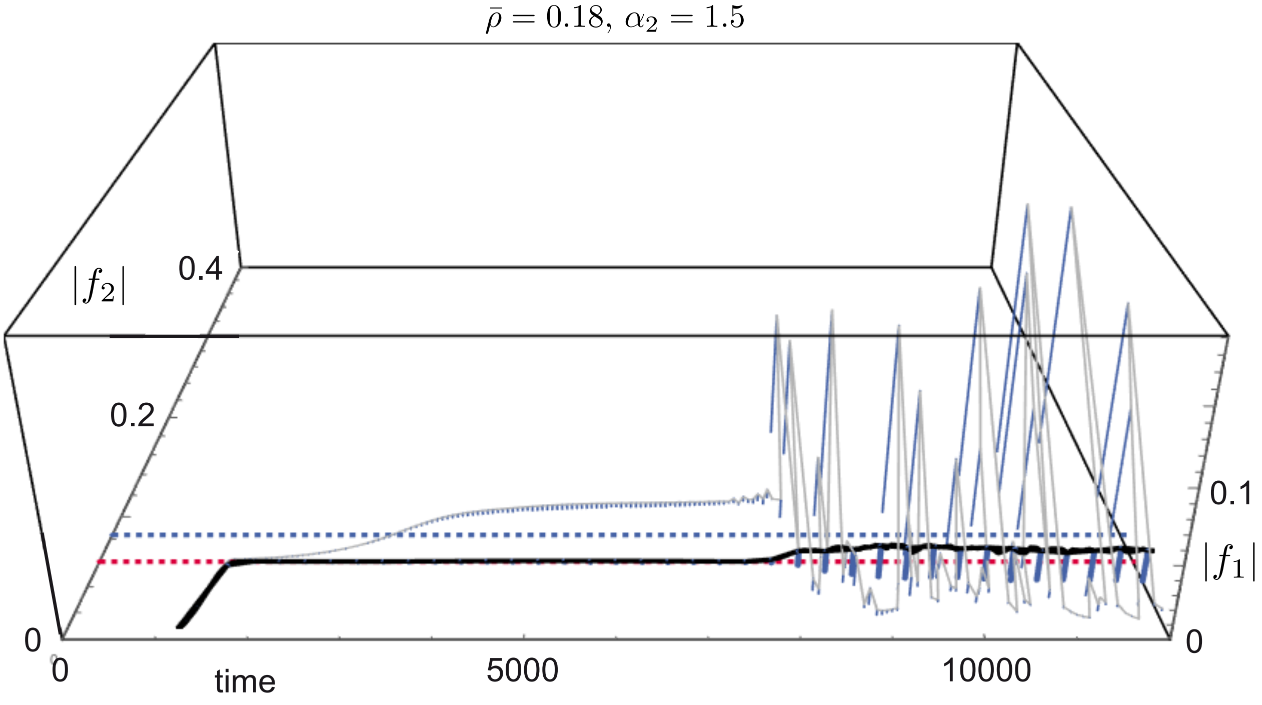

Fig. S7. Polar instability in generalized hydrodynamic equations. Evolution of the nematic and polar order of the hydrodynamic equations Eq. (9) with coefficients Eq. (11), $r\bar{h}o = 0.18$ and $\alpha = 1.5$. Solutions were obtained with XMDS software(15) as detailed in B for a system of size 800×800 with 250 lattice points per dimension. As initial condition we used a disordered state with small non-uniform fluctuations. The gray line shows the time trace of nematic and polar order parameters (respectively $|f_2|$ and $|f_1|$) close to the core of a nematic band. The average nematic and polar order are shown by the black line. The value of the polar amplitude $|f_1|$ along the time trace is indicated by blue bars to clearly visualize the growth of polar order. The red dashed line indicates the nematic order of the uniform solution. The blue dashed line indicates the nematic order of the uniform solution of Eqs. Eq. (9) at the critical density $\rho^{(c,h)}_{\text{nem-pol}}$. Subsequent to the formation of a nematic band, the nematic order within the band (grey line) eventually exceeds the value corresponding to $\rho^{(c,h)}_{\text{nem-pol}}$. After some time polar order within the band (vertical component of the grey line) and average polar order (vertical component of the black line) start to grow. For the final patterns, see Movie S4

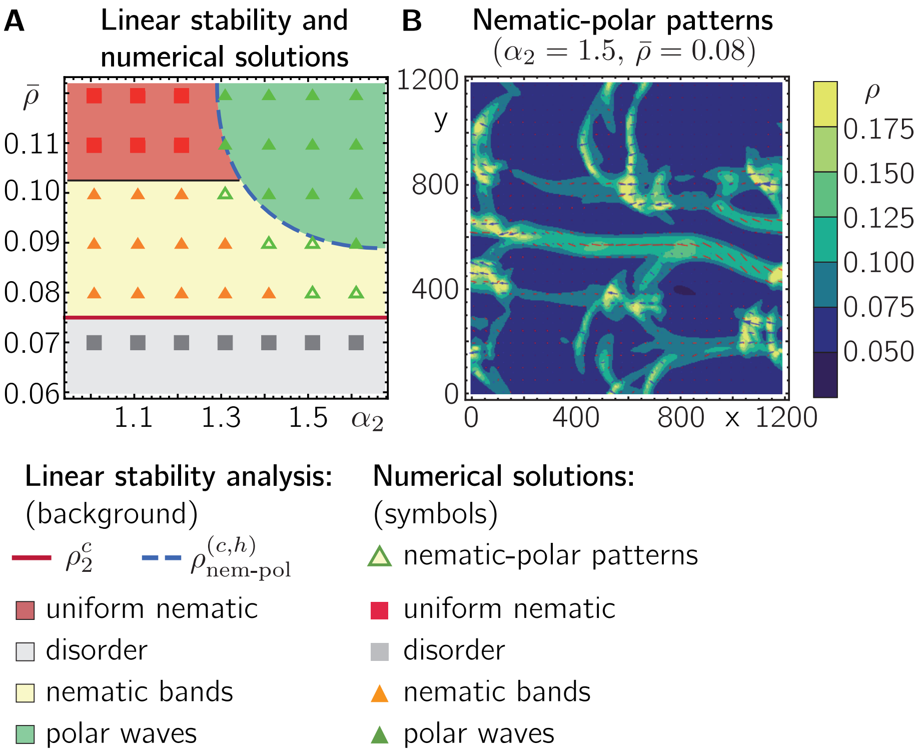

Fig. S8. Linear stability analysis of Eq. (9) **and coexistence patters with coefficients given by** Eq. (12)**. (A)** The predictions from linear stability analysis are given as background colors [as in Fig. 4(A)]. In particular, similarly as in Fig. 4(A), we find a direct transition, $\rho_{\text{nem-pol}}^{(c,h)}$, between a regime of nematic patterns (yellow background) and polar order (green background). Close to this transition, $\rho_{\text{nem-pol}}^{(c,h)}$, numerical solutions [symbols, as in Fig. 3(A)] reveal a regime of patterns with intertwined polar and nematic order (nematic-polar patterns). For numerical solutions we used `XMDS2` (15) software and a system size of 200 on a 80×80 lattice. **(B)** Snapshot of the numerical solution of Eq. (9) with the coefficients Eq. (12) in the regime of 'nematic-polar patterns' ($\alpha = 1.5, \bar{\rho} = 0.08$). For the numerical solution we used `XMDS2` (15) software and a system size of 1200 on a 260×260 lattice. The color code denotes the local densities, red bars and blue arrows show the orientation of local nematic and polar order, respectively (the length indicates their respective absolute amplitudes).

Movie S1. Transformations between nematic bands and polar waves. Parameters are $\bar{\rho} = 0.292$ and $\psi = 0.1\pi$. Results were obtained based on a modified version of the `SNAKE` algorithm on a 100×100 lattice with spacing 8, time step 0.3 and 40 angular slices. The system started from isotropic disorder with small random fluctuations with a seed different to the seed in Movies 2 and 3. The color shows the local density according to the color bar and the axis denote the extensions in units of the lattice spacing.

Movie S2. Nematic bands transform into polar waves. Parameters are $\bar{\rho} = 0.285$ and $\psi = 0.1\pi$. Results were obtained based on a modified version of the `SNAKE` algorithm on a 100×100 lattice with spacing 8, time step 0.3 and 40 angular slices. Initial conditions were chosen uniform disordered with small random fluctuations with a seed different to the seed in Movies 1 and 3. The color shows the local density according to the color bar and the axis denote the extensions in units of the lattice spacing.

Movie S3. Nematic bands transform into polar waves and back. Parameters are $\bar{\rho} = 0.285$ and $\psi = 0.1\pi$. Results were obtained based on a modified version of the `SNAKE` algorithm on a 60×60 lattice with spacing 8, time step 0.3 and 40 angular slices. Initial conditions were chosen uniform disordered with small random fluctuations with a seed different to the seed in Movies 1 and 2. The color shows the local density according to the color bar and the axis denote the extensions in units of the lattice spacing.

Movie S4. Coexistence and transformations of nematic bands and polar waves in generalized hydrodynamic equations. Parameters are $\bar{\rho} = 0.18$, $\alpha_2 = 1.5$. The color denotes the local density as given in the colour bar. Numerical solutions were obtained with XMDS software(15) as detailed in B for a system of size 800×800 with 250 lattice points per dimension. The simulation was initiated from a uniform disordered state with small random perturbations and the movie starts after the initial formation of nematic bands. The color shows the local density according to the color bar and the axis denote the spatial extensions.

References

1. E Bertin, M Droz, G Grégoire, Boltzmann and hydrodynamic description for self-propelled particles. *Phys. Rev. E* **74**, 022101 (2006).
2. E Bertin, M Droz, G Grégoire, Hydrodynamic equations for self-propelled particles: Microscopic derivation and stability analysis. *J. Phys. A: Math. Theor.* **42** (2009).
3. A Peshkov, IS Aranson, E Bertin, H Chaté, F Ginelli, Nonlinear field equations for aligning self-propelled rods. *Phys. review letters* **109**, 268701 (2012).
4. J Denk, L Huber, E Reithmann, E Frey, Active curved polymers form vortex patterns on membranes. *Phys. review letters* **116**, 178301 (2016).
5. L Huber, T Krüger, E Frey, Microphase separation in active filament systems is maintained by cyclic dynamics of cluster size and order. arxiv:2007.00517 (1 Jul 2020).
6. KDNT Lam, M Schindler, O Dauchot, Self-propelled hard disks: implicit alignment and transition to collective motion. *New J. Phys.* **17**, 113056 (2015).
7. KDNT Lam, M Schindler, O Dauchot, Polar active liquids: a universal classification rooted in nonconservation of momentum. *J. Stat. Mech. Theory Exp.* **2015**, P10017 (2015).
8. F Thüroff, CA Weber, E Frey, Numerical treatment of the boltzmann equation for self-propelled particle systems. *Phys. Rev. X* **4**, 041030 (2014).
9. B Mahault, Ph.D. thesis (2018).
10. H Chaté, Dry Aligning Dilute Active Matter. *Annu. Rev. Condens. Matter Phys.* **11** (2020).

11. AP Solon, H Chaté, J Tailleur, From phase to microphase separation in flocking models: The essential role of nonequilibrium fluctuations. *Phys. review letters* **114**, 068101 (2015).
12. MC Marchetti, et al., Hydrodynamics of soft active matter. *Rev. Mod. Phys.* **85**, 1143 (2013).
13. A Peshkov, E Bertin, F Ginelli, H Chaté, Boltzmann-Ginzburg-Landau approach for continuous descriptions of generic Vicsek-like models. *Eur. Phys. Journal: Special Top.* **223**, 1315–1344 (2014).
14. D Saintillan, MJ Shelley, Active suspensions and their nonlinear models. *Comptes Rendus Physique* **14**, 497–517 (2013).
15. GR Dennis, JJ Hope, MT Johnsson, Xmds2: Fast, scalable simulation of coupled stochastic partial differential equations. *Comput. Phys. Commun.* **184**, 201 – 208 (2013).
16. L Huber, R Suzuki, T Krüger, E Frey, A Bausch, Emergence of coexisting ordered states in active matter systems. *Science* **361**, 255–258 (2018).
